# Supplementary material for: Decoupling the Roles of Cell Shape and Mechanical Stress in Orienting and Cueing Epithelial Mitosis
Source: Cell Rep. 2019 Feb 19;26(8):2088–2100.e4. doi: 10.1016/j.celrep.2019.01.102 (PMC6381790; doi:10.1016/j.celrep.2019.01.102)
Supplement: Document S1. Methods S1 and Figures S1–S4 [file mmc1.pdf]

Cell Reports, Volume *26*

Supplemental Information

Decoupling the Roles of Cell Shape and Mechanical Stress in Orienting and Cueing Epithelial Mitosis

Alexander Nestor-Bergmann, Georgina A. Stooke-Vaughan, Georgina K. Goddard, Tobias Starborg, Oliver E. Jensen, and Sarah Woolner

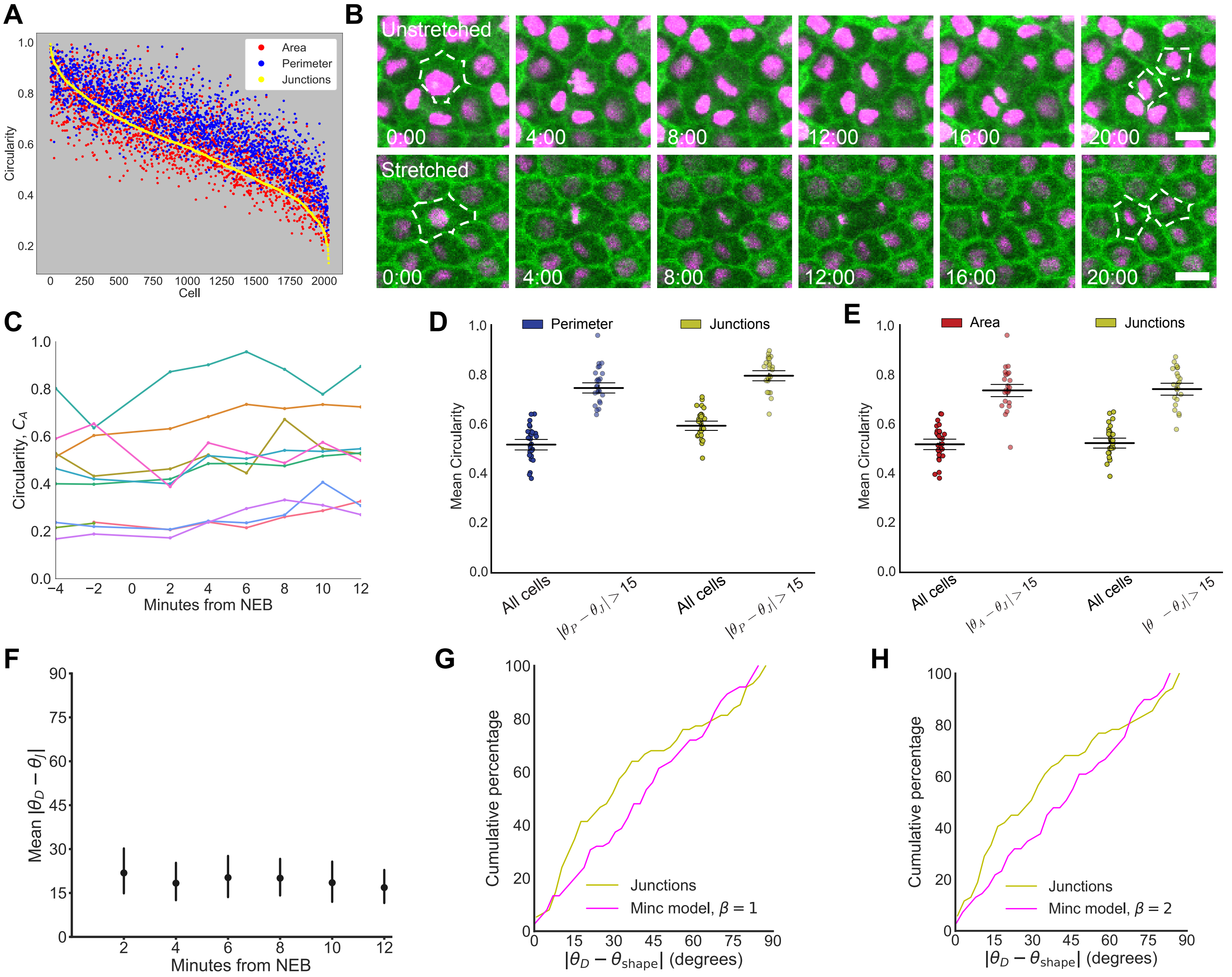

Figure S1. Related to Figure 2. A. Circularities of 2035 cells from unstretched experiments, with shape characterised by area, perimeter and junctions plotted in red, blue and yellow respectively. Cells have been ordered in descending order of junction-based circularity (C_J), with the corresponding values of C_A and C_P plotted alongside. **B.** Images taken from a confocal timelapse movie of a division in unstretched (upper) and stretched (lower) tissue from NEB to formation of cleavage plane. Cells do not show any significant rounding during this time period. **C.** Line plots showing circularity defined by cell area, C_A, against time (in minutes) since NEB for 9 example cells from stretched tissue. Lines show no significant trend of increasing or decreasing circularity. **D.** Mean circularity of subpopulation used in Figure 3E (darker points) vs mean circularity of all cells (lighter points), where shape is characterised by perimeter (blue) and junctions (yellow). Error bars represent mean and 95% confidence intervals. **E.** Equivalent plot to D, but shape characterised by area in red and junctions in yellow. **F.** Alignment of division orientation with junctional shape at different timepoints (minutes from NEB; n = 162 cells). **G.** Cumulative plot of difference between division angle, θ_D, and orientation of shape based on the Minc model when $\beta = 1$ (magenta; $\theta_{shape} = \theta_{Minc}$; Minc et al., 2011) and junctions (yellow; $\theta_{shape} = \theta_J$). **H.** As G but $\beta = 2$.

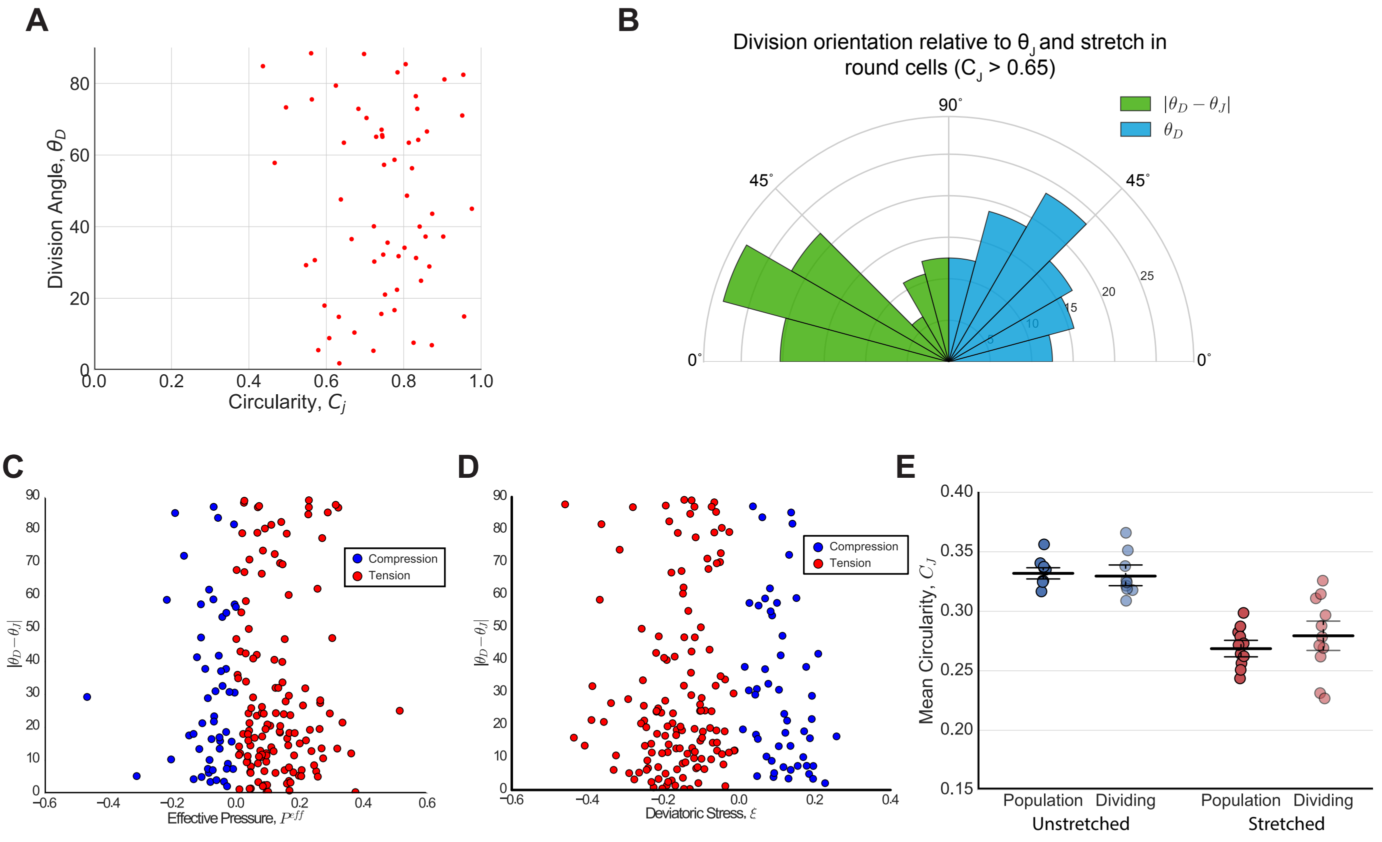

Figure S2. Related to Figure 3: A. Scatter plot of division angle relative to stretch direction (θ_D) vs cell circularity, C_J, showing no significant relationship. **B.** Rose histogram showing division orientation relative to TCJ shape ($|\theta_D - \theta_J|$) and the stretch axis (θ_D) in round cells ($C_J > 0.65$). **C.** Correlation of effective pressure vs $|\theta_D - \theta_J|$. Spearman rank correlation coefficient found no significant correlation. Cells under tension (compression) plotted in red (blue). **D.** Correlation of shear stress vs $|\theta_D - \theta_J|$. Spearman rank correlation coefficient found no significant correlation. Cells under tension (compression) plotted in red (blue). **E.** Comparison of mean circularity, C_J, of population of all cells vs dividing cells from unstretched and stretched control experiments. Error bars represent mean and 95% confidence intervals, which overlap between the population and dividing cells, indicating no significant difference.

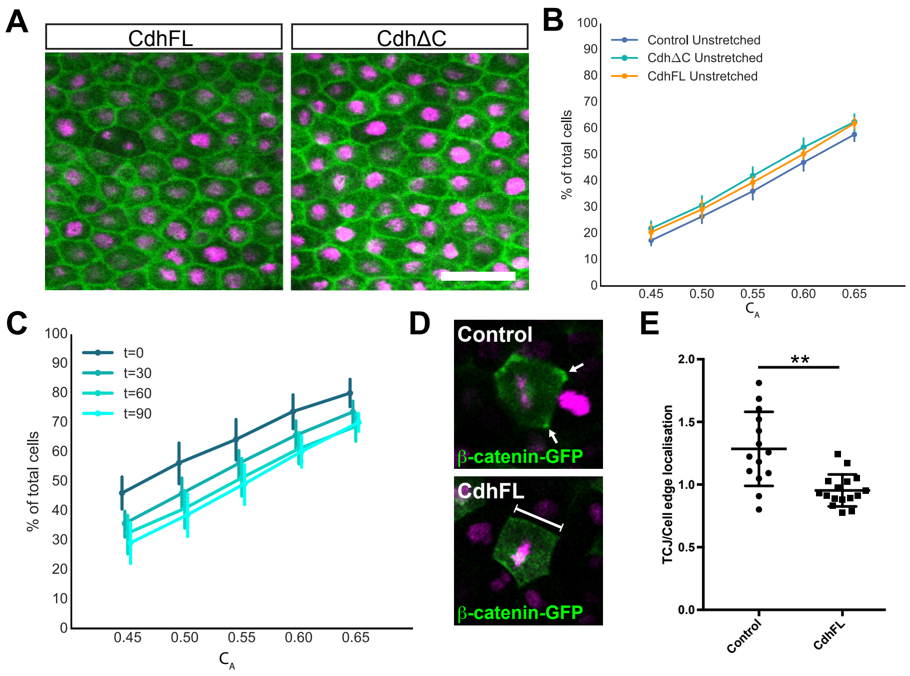

Figure S3. Related to Figure 4: A. Images taken from a confocal timelapse movie of CdhFL- (left) and CdhΔC- (right) injected stretched animal cap explants (green: GFP-alpha-tubulin; magenta: cherry-histone2B). Scale bar 50µm. **B.** Cumulative plots of cell circularity defined by area, C_A, in unstretched CdhΔC-injected (cyan), CdhFL-injected (orange) and control (blue) animal caps. Error bars represent 95% confidence intervals, which overlap indicating no significant difference. **C.** Cumulative plots of cell circularity defined by area, C_A, in CdhΔC-injected stretched animal caps at 0, 30, 60 and 90 mins after stretch (stretch applied just before 0 min). 100% of cells have $C_A < 1$. **D.** Image taken from time-lapse movies of control and CdhFL-injected animal cap tissue expressing β-catenin-GFP in a mosaic fashion. In control cells, β-catenin-GFP is enriched at TCJs during mitosis (arrows). The enrichment of β-catenin-GFP at TCJs is lost when CdhFL is expressed, with localisation spread throughout the cell edge (line). F. Quantification of β-catenin-GFP localization at TCJs compared to cell edges in single mitotic cells in animal caps. β-catenin-GFP is more strongly localised at TCJs compared to cell edges in controls but this bias is lost in CdhFL-injected tissue (*$p < 0.01$ Komogorov-Smirnov test; n = 14, 16 mitotic cells from 6, 4 unrelated animal caps for control and CdhFL, respectively).

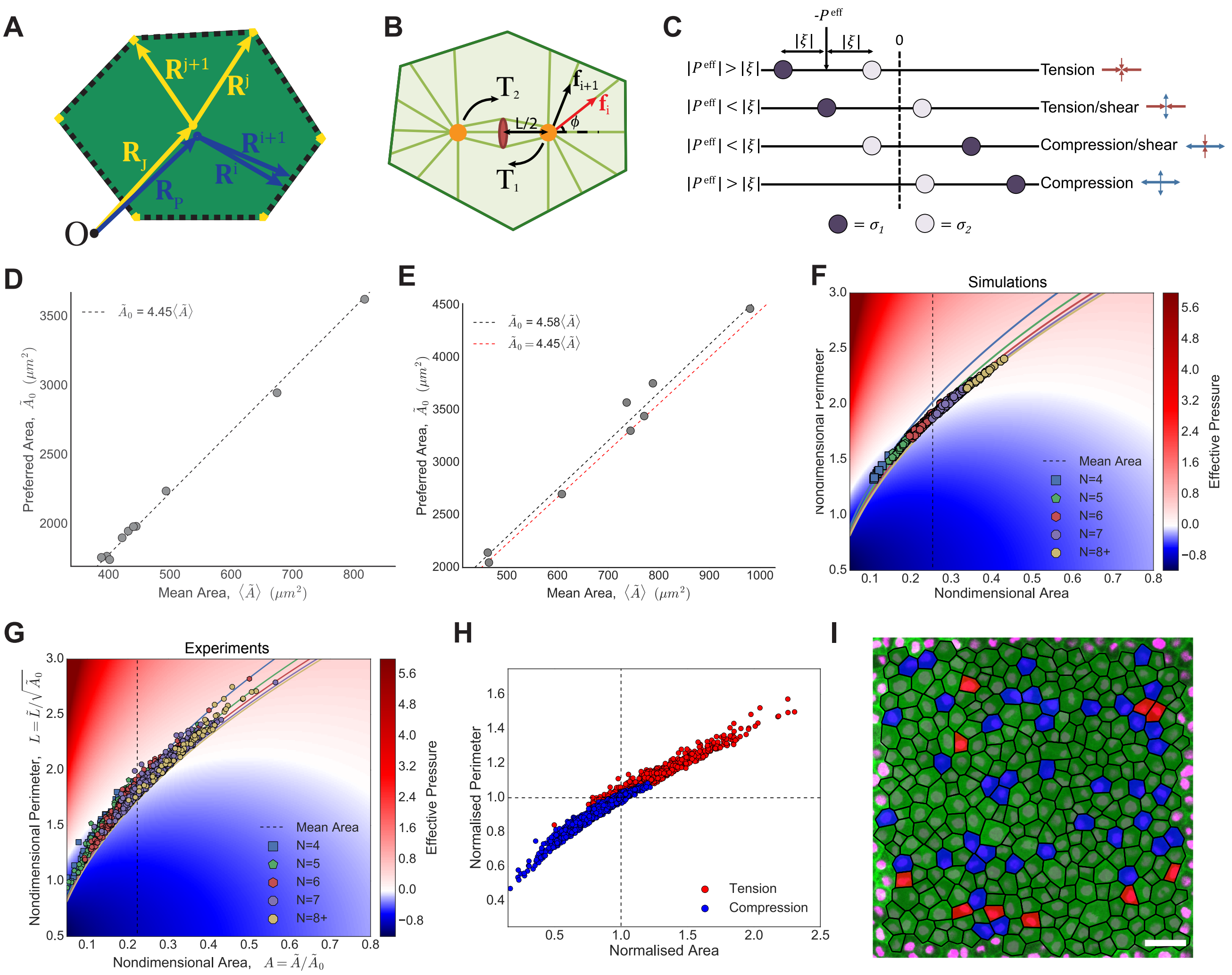

Figure S4. Related to STAR Methods: A. Representation of cell geometry outlined in STAR methods and Methods S1. Cells are computationally segmented such that their boundary pixels (black dots) and tricellular junctions (yellow dots) are extracted. Vectors based on cell perimeter (tricellular junctions) given in blue (yellow), with the centroid labeled R_P, (R_J). **B.** Shape-based division model proposed by Minc et al., 2011. Microtubules (light green lines) exert length-dependent forces on the spindle about the centrosomes (orange dots), exerting a net torque and rotating the spindle. **C**.Four possible cases of cell stress, based on the eigenvalues of the stress tensor. Horizontal lines represent a shared real number line with dark (light) circles indicating the value of σ_1 (σ_2). Vertical dashed line marks the location of zero. Arrows on far right represent direction of principal axes of stress, with red (blue) lines indicating tension (compression). **D**. Mean cell areas, $\langle\tilde{A}\rangle$, from 10 animal cap explants plotted against the preferred area, $\tilde{A}_0$, fitted to each experiment. Cells were polygonised (straight edges drawn between tricellular junctions) before the areas were computed, in order to be equivalent to the vertex-based model. Dashed line indicates linear line of best fit. **E.** Mean cell areas, $\langle\tilde{A}\rangle$, plotted against the preferred area, $\tilde{A}$, from 8 myosin II MO animal cap explants. Black (red) dashed line indicates linear line of best fit for myosin II MO (control, given in D) experiments. ANCOVA test suggests that slope of myosin II MO cells is not significantly different to control. **F.** Heat map showing relative effective pressure, P^{eff}, of a realisation of 800 simulated cells in a periodic box, with parameters (Λ,Γ) = (-0.259, 0.172), under conditions of zero global stress. Cell polygonal class indicated by marker colour and style, with (4,5,6,7,8+) sided cells given in (blue, green, red, purple, yellow). Equivalently coloured lines indicate exact perimeter-area relationship for perfect polygons. Dashed vertical line represents mean area of all cells. Cells lying in red (blue) regions are under net tension (compression). **G.** Equivalent plot to F for experimental control unstretched population data. Cell areas and perimeters have been nondimensionalised using the preferred area, $\tilde{A}_0$, such that the relative effective pressure may be calculated. A separate $\tilde{A}_0$ was fitted to each experiment, to account for differences in average cell size across experiments (data shown in Figure S4D). **H.** Normalised area vs normalised perimeter for the experimental cells given in G. Cell area and perimeter were normalised relative to the mean of each experiment. Cells under net tension (compression) are plotted in red (blue). Dashed vertical (horizontal) line represents cell area (perimeter) equal to the mean. **I.** Example segmented cells from an unstretched experiment, demonstrating where area is a poor predictor of effective pressure. Cells in red (blue) are predicted to be under tension (compression), but have area smaller (larger) than the mean. Scale bar 50μm.

Methods S1. Related to STAR Methods

1 Deriving cell shape tensors

We characterise cell shape by deriving three shape tensors based on the distribution of cell area, perimeter and tricellular junctions. The shape tensors are defined via the cells' second moments of area, perimeter or tricellular junctions and so each is associated with a different centre of mass, which is defined in terms of the first moments of area, perimeter or tricellular junctions respectively. We provide equations for calculating the centroid using each measure; the centroid may also be calculated as the arithmetic mean position of all the points used to define each measure.

Cells from the confocal imaging data are computationally segmented, giving N_C cells labelled with greek subscripts $\alpha = 1, 2, \ldots, N_C$. Each cell is defined by P pixels, labelled anticlockwise $i = 0, 1, \ldots, P-1$, representing the cell cortex, with Z tricellular junctions (pixels shared by three cells; we include the rare cases in which pixels are shared by more than three cells), labelled anticlockwise $j = 0, 1, \ldots, Z-1$. Boundary pixels are labelled with Latin super/sub-scripts, i, and tricellular junctions with Latin super/sub-scripts, j. Subscripts denote vectors originating from the origin (i.e. $\mathbf{R}_i$) and superscripts denote vectors originating from cell centroids (i.e. $\mathbf{R}^i$). Tangents between pixels are denoted $\mathbf{t}^i = \mathbf{R}^{i+1} - \mathbf{R}^i$. An image detailing the geometry of the system is given in Supplemental Figure 4A.

1.1 Tricellular junction-based centroid and shape tensor

The tricellular junction-based centroid, $\mathbf{R}_J$, is the arithmetic mean position of the cell's tricellular junctions. For a cell with Z tricellular junctions labelled $j = 0, \ldots, Z-1$ we have

$$\mathbf{R}_J = \frac{1}{Z} \sum_{j=0}^{Z-1} \mathbf{R}_j \tag{1.1}$$

where $\mathbf{R}_j$ is the vector running from the origin to tricellular junction j.

Let $\mathbf{R}^j = \mathbf{R}_j - \mathbf{R}_J$ be the vector running from the cell centroid to tricellular junction j. We define the tricellular junction-based shape tensor as

$$\mathsf{S}_J = \frac{1}{Z} \sum_{i=0}^{Z-1} \mathbf{R}^i \mathbf{R}^i, \tag{1.2}$$

where $\mathbf{R}^i \mathbf{R}^i$ is a vector outer product.

1.2 Perimeter-based centroid and shape tensor

The perimeter-based centroid, $\mathbf{R}_P$, of cell α, is classically defined as

$$\mathbf{R}_P = \frac{\int_{\partial\alpha} \mathbf{R}\, \mathrm{d}s}{\int_{\partial\alpha} \mathrm{d}s}, \tag{1.3}$$

where the numerator is the first moment of perimeter, $\mathbf{R}$ represents vectors across the continuous cell perimeter from the origin, $\mathrm{d}s$ is a line element and the integral is around the cell perimeter, $\partial\alpha$. For a polygonised segmented cell, we can write

$$\mathbf{R} = \mathbf{R}^i + b\mathbf{t}^i \tag{1.4}$$

and

$$\mathrm{d}s = |\mathbf{t}^i|\,\mathrm{d}b = l^i\,\mathrm{d}b, \tag{1.5}$$

where $0 \le b \le 1$ and l^i is the length of the straight line connecting pixels i and $i+1$. Thus (1.3) becomes

$$\begin{aligned}\mathbf{R}_P &= \frac{\sum\limits_{i=0}^{Z-1} \int_0^1 \left[\mathbf{R}^i + b(\mathbf{R}^{i+1} - \mathbf{R}^i)\right] l^i\,\mathrm{d}b}{\sum\limits_{i=0}^{Z-1} l^i\,\mathrm{d}b} \\ &= \frac{1}{L}\sum_{i=0}^{Z-1} \frac{l^i}{2}\left(\mathbf{R}^i + \mathbf{R}^{i+1}\right), \end{aligned} \tag{1.6}$$

where $L = \sum\limits_{i=0}^{Z-1} l^i$ is the perimeter of the cell.

We define the perimeter-based shape tensor, S_P, via the second moment of perimeter as

$$\mathsf{S}_P = \int_{\partial\alpha} (\mathbf{R} - \mathbf{R}_P)(\mathbf{R} - \mathbf{R}_P)\,\mathrm{d}s. \tag{1.7}$$

Using (1.4) and (1.5), this becomes

$$\begin{aligned}\mathsf{S}_P &= \sum_{i=0}^{P-1} \int_0^1 \left[\mathbf{R}^i\mathbf{R}^i + b(\mathbf{t}^i\mathbf{R}^i + \mathbf{R}^i\mathbf{t}^i) + b^2\mathbf{t}^i\mathbf{t}^i\right] l^i\,\mathrm{d}b \\ &= \sum_{i=0}^{P-1} \left[\mathbf{R}^i\mathbf{R}^i + \tfrac{1}{2}(\mathbf{t}^i\mathbf{R}^i + \mathbf{R}^i\mathbf{t}^i) + \tfrac{1}{3}\mathbf{t}^i\mathbf{t}^i\right] l^i \\ &= \sum_{i=0}^{P-1} l^i \left[\tfrac{1}{3}(\mathbf{R}^{i+1}\mathbf{R}^{i+1} + \mathbf{R}^i\mathbf{R}^i) + \tfrac{1}{6}(\mathbf{R}^{i+1}\mathbf{R}^i + \mathbf{R}^i\mathbf{R}^{i+1})\right]. \end{aligned} \tag{1.8}$$

1.3 Area-based centroid and shape tensor

We define the area-based centroid, $\mathbf{R}_A$, of cell α, via the cell's first moment of area

$$\mathbf{R}_A = \frac{\int_\alpha \mathbf{R}\,\mathrm{d}A}{\int_\alpha \mathrm{d}A}, \tag{1.9}$$

where the numerator is the first moment of area, $\mathbf{R}$ represents vectors across the cell area from the origin, $\mathrm{d}A$ is an area element and the integral is across the cell interior. This is the classically defined centroid, equivalent to the centre of a best-fit ellipse. The integral can be evaluated using Green's theorem and, assuming the cell is a convex polygon, the formula may be discretised to give

$$\mathbf{R}_A = \frac{1}{6A}\sum_{i=0}^{P-1}[\mathbf{R}^i + \mathbf{R}^{i+1}][\hat{\mathbf{z}}\cdot(\mathbf{R}^i \times \mathbf{R}^{i+1})], \tag{1.10}$$

where $A = \sum_{i=0}^{P-1} \frac{1}{2}\hat{\mathbf{z}}\cdot(\mathbf{R}^i \times \mathbf{R}^{i+1})$ is the area of the polygon and $\hat{\mathbf{z}}$ is a unit vector pointing out of the plane.

To define the area-based shape tensor, let us consider the triangle, $\Delta^i = \{\mathbf{R}_A, \mathbf{R}^i, \mathbf{R}^{i+1}\}$, and define

$$\mathbf{R} = \mathbf{R}_A + a\mathbf{R}^i + b\mathbf{t}^i, \tag{1.11}$$

for $\mathbf{R}$ in Δ^i. The edges of the triangle are then given by

$$b = 0, \qquad 0 < a < 1 \tag{1.12a}$$
$$a = 1, \qquad 0 < b < 1 \tag{1.12b}$$
$$b = a, \qquad 0 < a < 1. \tag{1.12c}$$

Computing the second moment of the triangle, Δ^i, we have

$$\begin{aligned}
\mathsf{S}_{\Delta^i} &= \int_{\Delta^i} (\mathbf{R} - \mathbf{R}_A)(\mathbf{R} - \mathbf{R}_A)\,\mathrm{d}A. \\
&= \int_{\Delta^i} (a\mathbf{R}^i + b\mathbf{t}^i)(a\mathbf{R}^i + b\mathbf{t}^i)\,\mathrm{d}A \\
&= \int_{\Delta^i} \left[a^2\mathbf{R}^i\mathbf{R}^i + ab(\mathbf{R}^i\mathbf{t}^i + \mathbf{t}^i\mathbf{R}^i) + b^2\mathbf{t}^i\mathbf{t}^i\right]\mathrm{d}A.
\end{aligned} \tag{1.13}$$

Taking an area element of the triangle gives

$$\begin{aligned}
\mathrm{d}A &= \left|\frac{\partial(x,y)}{\partial(a,b)}\right| \mathrm{d}a\,\mathrm{d}b \\
&= \left|\hat{\mathbf{z}} \cdot \mathbf{R}^i \times \mathbf{t}^i\right| \mathrm{d}a\,\mathrm{d}b.
\end{aligned} \tag{1.14}$$

Integrating over the triangle, we have

$$\begin{aligned}
\int_{\Delta^i} \mathrm{d}A &= \int_0^1 \mathrm{d}a \int_0^a \mathrm{d}b \left|\hat{\mathbf{z}} \cdot \mathbf{R}^i \times \mathbf{t}^i\right| \\
&= \tfrac{1}{2}\left|\hat{\mathbf{z}} \cdot \mathbf{R}^i \times \mathbf{t}^i\right| \\
&= A^i,
\end{aligned} \tag{1.15}$$

where A^i is the area of Δ^i. Thus

$$\begin{aligned}
\mathsf{S}_{\Delta^i} &= \mathbf{R}^i\mathbf{R}^i \int_0^1 \mathrm{d}a \int_0^a a^2 2A^i\,\mathrm{d}b + \mathbf{t}^i\mathbf{t}^i \int_0^1 \mathrm{d}a \int_0^a b^2 2A^i\,\mathrm{d}b \\
&\quad + (\mathbf{R}^i\mathbf{t}^i + \mathbf{t}^i\mathbf{R}^i) \int_0^1 \mathrm{d}a \int_0^a ab2A^i\,\mathrm{d}b \\
&= A^i\left[\tfrac{1}{2}\mathbf{R}^i\mathbf{R}^i + \tfrac{1}{6}\mathbf{t}^i\mathbf{t}^i + \tfrac{1}{4}(\mathbf{R}^i\mathbf{t}^i + \mathbf{t}^i\mathbf{R}^i)\right] \\
&= \frac{A^i}{6}(\mathbf{R}^{i+1}\mathbf{R}^{i+1} + \mathbf{R}^i\mathbf{R}^i) + \frac{A^i}{12}(\mathbf{R}^{i+1}\mathbf{R}^i + \mathbf{R}^i\mathbf{R}^{i+1}).
\end{aligned} \tag{1.16}$$

Finally, the area-based shape tensor, S_A, can be defined as the sum of the second moments of each sub-triangle:

$$\begin{aligned}
\mathsf{S}_A &= \int_\alpha (\mathbf{R} - \mathbf{R}_A)(\mathbf{R} - \mathbf{R}_A)\,\mathrm{d}A \\
&= \sum_{i=0}^{Z-1} \mathsf{S}_{\Delta^i} \\
&= \sum_{i=0}^{Z-1} \left\{\frac{A^i}{6}(\mathbf{R}^{i+1}\mathbf{R}^{i+1} + \mathbf{R}^i\mathbf{R}^i) + \frac{A^i}{12}(\mathbf{R}^{i+1}\mathbf{R}^i + \mathbf{R}^i\mathbf{R}^{i+1})\right\}.
\end{aligned} \tag{1.17}$$

1.4 Cell circularity and orientation

The cell shape tensors, S_X (for $X \in \{J, P, A\}$), have eigenvalues $\lambda_{X,1} \geq \lambda_{X,2}$. We define the X-based cell circularity as

$$C_X = \frac{\lambda_{X,2}}{\lambda_{X,1}}, \tag{1.18}$$

such that $0 \geq C_X \geq 1$ and C_X is larger for more rounded cells. The orientation of X-based cell shape, θ_X, may then be defined as the orientation of the eigenvector associated with the largest eigenvalue.

2 Predicting division orientation using a mechanical model based on microtubule pulling forces

The geometric shape-based predictions of division were compared against a mechanical shape-based model of cell division, in which astral microtubules are hypothesised to exert pulling forces on the spindle (Minc et al., (2011)). The spindle is assumed to lie in the 2D apical plane of the cell, centred at the cell centroid, $\mathbf{R}_P$ (choosing $\mathbf{R}_P$ to account for non-straight edges). Microtubules (MTs) emanate from the opposed centrosomes of the spindle in a straight line towards the cortex. Anchoring at the cell cortex, the MTs are assumed to exert length-dependent forces towards the centrosome. This leads to a torque on the spindle about the centroid, dependent on the angle, ϕ, between the MT and the spindle axis. The configuration can be visualised in Supplemental Figure 4B.

Let the spindle, of length L, be oriented at angle $\theta \in [0, \pi]$ relative to a reference axis. We create two asters at each end of the spindle, labelled $j = 1, 2$ and positioned at $(-L/2, L/2)$. Each aster has N MTs of length $l_i(\phi, \theta)$, placed at a constant angular density. A MT oriented at angle ϕ relative to the spindle axis exerts a length-dependent force of magnitude

$$f_i(\phi, \theta) = l_i^{\beta}(\phi, \theta) \cos\phi \tag{2.1}$$

on the spindle, along the axis of the microtubule. β is a variable governing how the force exerted by a MT scales with its length. Correspondingly, the torque exerted on the spindle by the MT is

$$\tau_i(\phi, \theta) = \frac{L}{2} l_i^{\beta}(\phi, \theta) \sin\phi. \tag{2.2}$$

The total torque, T, exerted by aster j on the spindle is then given by

$$T_j(\theta) = \int_{-\phi_{\text{max}}}^{\phi_{\text{max}}} \rho \tau_i(\phi, \theta) \, \mathrm{d}\phi \tag{2.3}$$

where ϕ_{max} is the maximum angle that a MT makes relative to the spindle, such that $2\phi_{\text{max}}$ is the angular width of the aster, and $\rho = 2\phi_{\text{max}}/N$ is the constant angular density. The net torque exerted on the spindle is the sum of torques from each aster:

$$T_S(\theta) = T_1(\theta) + T_2(\theta) \tag{2.4}$$

(2.4) can be computed for $\theta \in [0, \pi]$ and the cell's predicted division angle, θ_{Minc} is given by the angle at which the net torque is minimised i.e.

$$\theta_{\text{Minc}} = \underset{\theta \in [0,\pi]}{\arg\min}\, T_S(\theta). \tag{2.5}$$

Zeros of (2.4) usually come in pairs, one stable and one unstable, and we choose the stable solution. For the subset of cases where (2.4) has multiple stable zeros (usually occurring in round cells), we choose the solution producing the lowest minimum of the derivative.

The model has parameters $(L, N, \phi_{\max}, \beta)$, corresponding to length of the spindle, number of MTs at each aster, angular extension of the MTs and the force-length scaling parameter. However, previous studies with this model have found that a number of these parameters become redundant beyond a certain value (Minc et al. (2011), Bosveld et al. (2016)). Bosveld et al. (2016) varied the spindle length, L, to 1/8 of the experimentally measured value and found no discernible change in the predicted division angles. Following average spindle length measurements by Woolner et al. (2008), we therefore set $L = \sqrt{A/\pi}$, where A is the area of the cell, using the length of the cell fitted to a circle as the default spindle length. Similarly, choosing values of $N > 15$ and $\phi_{\max} > 120$ did not change overall predictions, so we set $N = 100$ and $\phi_{\max} = 180$ for all experiments. Conversely, varying β can produce discernible differences, with $\beta = 3$ producing the best prediction for Minc et al. (2011). We therefore followed Minc and colleagues by calculating all predictions for $\beta = 1, 2, 3$. Though predicted division angles differed slightly between these values, we found no significant difference when compared to predictions of division angle relative to the positioning of tricellular junctions (Figures 2I&J and S1G&H).

3 The vertex-based model

To model the cells mathematically, we adopt a well-established vertex-based constitutive model (Farhadifar et al., (2007), Ishihara et al., (2012), Nestor-Bergmann et al., (2017)). Under the vertex-based model, forces are obtained by assuming that every cell has a mechanical energy, $\tilde{U}$, of the following form

$$\tilde{U} = \tfrac{1}{2}\tilde{K}\left(\tilde{A} - \tilde{A}_0\right)^2 + \tfrac{1}{2}\tilde{\Gamma}\tilde{L}^2 + \tfrac{1}{2}\tilde{\Lambda}\tilde{L}, \tag{3.1}$$

where $\tilde{A}$ is the area of a cell, $\tilde{L}$ is the perimeter, $\tilde{A}_0$ is the preferred cell area, $\tilde{K}$ is a bulk stiffness and $(\tilde{\Lambda}, \tilde{\Gamma})$ are model parameters representing contractility of the cell periphery. We nondimensionalise, scaling length scales on $\sqrt{\tilde{A}_0}$, using

$$\tilde{A} = \tilde{A}_0 A, \quad \tilde{L} = \sqrt{\tilde{A}_0}L, \quad \tilde{U} = \tilde{K}\tilde{A}_0^2 U, \quad \tilde{t} = \eta t/(\tilde{K}\sqrt{\tilde{A}_0}), \tag{3.2}$$

where $\tilde{t}$ is time and η is a viscous drag coefficient. Thus the nondimensional mechanical energy, U, may be written

$$U = \tfrac{1}{2}\left(A_\alpha - 1\right)^2 + \tfrac{1}{2}\Gamma(L_\alpha - L_0)^2 - U_0 \tag{3.3}$$

where $U_0 = \Lambda^2/4\Gamma^2$ is a constant that may be discarded as the dynamics are driven by energy gradients and we have written $L_0 = -\Lambda/2\Gamma$ which defines an effective preferred cell perimeter. We are left with two nondimensional parameters

$$\Gamma = \frac{\tilde{\Gamma}}{\tilde{K}\tilde{A}_0}, \qquad \Lambda = \frac{\tilde{\Lambda}}{\tilde{K}\tilde{A}_0^{3/2}}, \tag{3.4}$$

which have previously been fitted to *Xenopus laevis* animal cap explants with values $(\Lambda, \Gamma) = (-0.259, 0.172)$ (see Nestor-Bergmann et al., (2017) for details on the fitting procedure). We assume that the cells have identical physical properties, such that (Λ, Γ) do not vary across the cells.

Under this constitutive equation, a cell can be mechanically characterised by its stress tensor, $\boldsymbol{\sigma}$, given by (Nestor-Bergmann et al., (2017) and Sugimura et al., (2016))

$$\boldsymbol{\sigma} = \overline{\boldsymbol{\sigma}} + \boldsymbol{\sigma}' - \frac{1}{2}\dot{\mathsf{S}}_J \tag{3.5}$$

where $\dot{\mathsf{S}}_J$ is the time derivative of the junctional shape tensor (1.2), which gives the dissipative stress acting when cells are out of equilibrium and arises from a simple choice of drag law on the vertices. We have written the elastic stress as a sum of isotropic and deviatoric contributions, with the former given by

$$\overline{\boldsymbol{\sigma}} = -P^{\text{eff}}\mathsf{I}, \qquad P^{\text{eff}} = A - 1 + \frac{\Gamma L^2}{2A} - \frac{\Lambda L}{4A}, \tag{3.6}$$

where I is the identity and P^{eff} is an effective cell pressure. The traceless contribution is

$$\boldsymbol{\sigma}' = \frac{\Gamma(L - L_0)}{A}\left(\frac{L}{2}\mathsf{I} - \sum_{j=0}^{Z-1} l^j \hat{\mathbf{t}}^j \hat{\mathbf{t}}^j\right) \tag{3.7}$$

where the cell has Z tricellular junctions, labelled anticlockwise $j = 0, 1, \ldots, Z-1$, and $\hat{\mathbf{t}}^j$ is the unit tangent connecting tricellular junctions j and $j+1$ and l^j is the length of that edge.

Assuming the cells are in equilibrium ($\dot{\mathsf{S}}_J = 0$), we can focus on the elastic contribution. Here the stress tensor will have eigenvalues

$$\sigma_1 = -P^{\text{eff}} + \xi \qquad \sigma_2 = -P^{\text{eff}} - \xi \tag{3.8}$$

such that $|\sigma_1| \geq |\sigma_2|$. $-P^{\text{eff}}$ is the eigenvalue of $\overline{\boldsymbol{\sigma}}$, by which we characterise the magnitude of isotropic stress. $\pm\xi$ are the eigenvalues of $\boldsymbol{\sigma}'$, with $\xi = \sqrt{\det(\boldsymbol{\sigma}')}$, representing the magnitude of shear stress. We term the eigenvector, $\boldsymbol{\sigma}_1$, associated with σ_1 the principal axis of stress. For $P^{\text{eff}} > 0$ (< 0) we have a cell dominantly under tension (compression). If $|\xi| > |P^{\text{eff}}|$, the cell is in a state of shear where the principal components of stress have opposite signs ($\sigma_1\sigma_2 < 0$). Supplemental Figure 4C outlines the four possible cases of cell stress, based on the signs of the eigenvalues.

4 Nondimensionalising the experimental data

The nondimensionalisation procedure uses the scalings on area and perimeter given in (3.2). In order to nondimensionalise the areas and perimeters from the experimental data, we must estimate the value of $\tilde{A}_0$ for the *Xenopus laevis* animal cap cells. Under (3.5), the isotropic component of the nondimensional tissue stress, P_{tis}, of a monolayer in equilibrium may be expressed as an area-weighted sum of the effective pressures (Nestor-Bergmann et al., (2017))

$$P_{\text{tis}} = \frac{1}{A_{\text{tis}}} \sum_{\alpha=1}^{N_c} A_\alpha P_\alpha^{\text{eff}} \tag{4.1}$$

where A_{tis} is the total surface area of the monolayer and the summation is over all cells labelled $\alpha = 1, \ldots, N_c$. Under conditions of zero external loading, $P_{\text{tis}} = 0$ and, substituting the scalings for area and perimeter in (3.2), we may write

$$\sum_{\alpha=1}^{N_c} \frac{\tilde{A}_\alpha}{\tilde{A}_0}\left(\frac{\tilde{A}_\alpha}{\tilde{A}_0} - 1 + \frac{\Gamma \tilde{L}_\alpha^2}{2\tilde{A}_\alpha} + \frac{\Lambda \tilde{L}_\alpha \sqrt{\tilde{A}_0}}{4\tilde{A}_\alpha}\right) = 0. \tag{4.2}$$

Thus, given previously fitted values of (Λ, Γ) and measurements of experimental areas, $\tilde{A}_\alpha$, and perimeters, $\tilde{L}_\alpha$, we can select the value of $\tilde{A}_0$ which best satisfies (4.2); see supplementary movie 1 for an example of the process). Given that the mean cell area varies between experiments (due to each embryo being at a slightly different stage of development when imaged) we fit a specific $\tilde{A}_0$ to every experiment. The implicit assumption – that the sample of experimental cells measured is under zero net peripheral stress – highlights that the results will be more accurate as the sample size increases.

Supplemental Figure 4D shows the mean cell areas, $\langle \tilde{A} \rangle$, from 10 experiments plotted against the fitted values of $\tilde{A}_0$. We see a strikingly linear relationship, indicating that the preferred area is well predicted by the mean area under our fitting process, where $\tilde{A}_0 \approx 4.45 \langle \tilde{A} \rangle$. These data also emphasise the variability in mean area across experiments, due the animal caps being at slightly different stages of development, indicating that each experiment requires a unique $\tilde{A}_0$. Supplemental Figure 4E shows the equivalent plot for myosin II MO cells, in which we see the cells have a significantly larger preferred area but $\tilde{A}_0 \approx 4.45 \langle \tilde{A} \rangle$ holds.

We can then infer the relative nondimensional effective pressure, for example, of the experimental cells by epressing nondimensional areas and perimeters in terms of the fitted $\tilde{A}_0$

$$P^{\text{eff}} = \frac{\tilde{A}}{\tilde{A}_0} - 1 + \frac{\Gamma \tilde{L}^2}{2\tilde{A}} + \frac{\Lambda \tilde{L} \sqrt{\tilde{A}_0}}{4\tilde{A}}, \tag{4.3}$$

where we have dropped the α subscripts. In the absence of a measurement of the stiffness parameter, K, (4.3) reveals relative stresses within and between cells in tissue, but not their absolute magnitude.

Supplemental Figures 4F&G demonstrate the relationship between cell area, perimeter and P^{eff}. Strikingly, we find that P^{eff} values calculated from both simulated (Supplemental Figure 4F) and experimental (Supplemental Figure 4G) data lie close to those predicted for perfect polygons, which have an exact relationship between area and perimeter (curves plotted in Supplemental Figures 4F&G). This indicates that the magnitude of isotropic cell stress is well predicted by area and number of sides. However, our results also demonstrate that cell area alone is not a perfect indicator of relative effective cell pressure. We find many cells have a negative (positive) value of P^{eff}, suggesting that they are under compression (tension), but have an area larger (smaller) than the mean (see blue cells above mean area and red cells below mean area in Supplemental Figure 4H). Examples of these special cases are given in Supplemental Figure 4I, where it is apparent that these cells do not look especially unusual.
