# Supplementary material for: Decoupling the Roles of Cell Shape and Mechanical Stress in Orienting and Cueing Epithelial Mitosis
Source: Cell Rep. 2019 Feb 19;26(8):2088–2100.e4. doi: 10.1016/j.celrep.2019.01.102 (PMC6381790; doi:10.1016/j.celrep.2019.01.102)
Supplement: Document S2. Article plus Supplemental Information [file mmc3.pdf]

Cell Reports

Article

# Decoupling the Roles of Cell Shape and Mechanical Stress in Orienting and Cueing Epithelial Mitosis

## Graphical Abstract

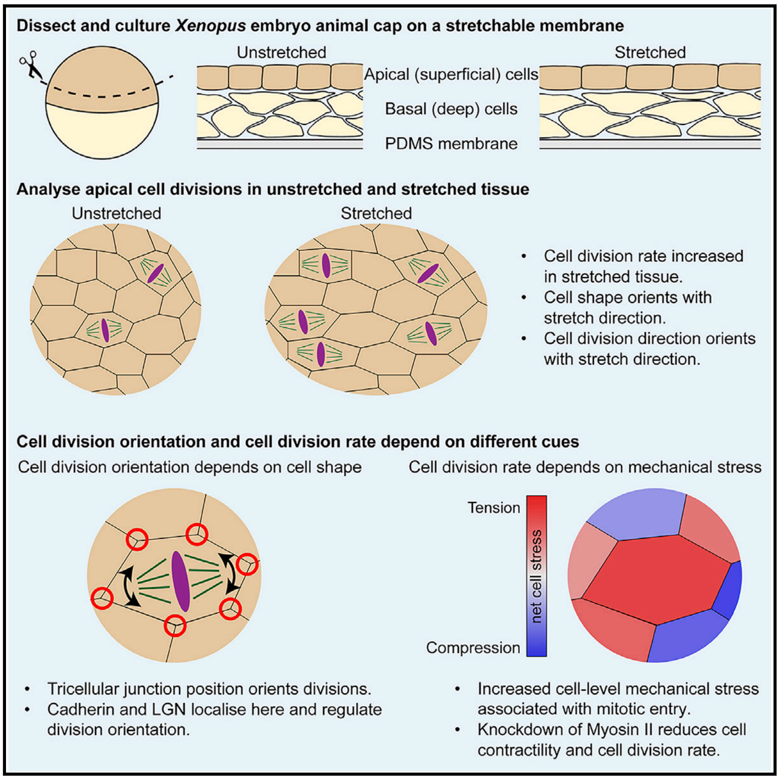


## Highlights

- Tissue stretching increases division rate and reorients divisions with stretch
- Division orientation is regulated by cell shape defined by tricellular junctions
- Cadherin and LGN localize to tricellular junctions aligning division to cell shape
- Division rate is linked to mechanical stress and can be decoupled from cell shape

## Authors

Alexander Nestor-Bergmann, Georgina A. Stooke-Vaughan, Georgina K. Goddard, Tobias Starborg, Oliver E. Jensen, Sarah Woolner

## Correspondence

an529@cam.ac.uk (A.N.-B.), sarah.woolner@manchester.ac.uk (S.W.)

## In Brief

Nestor-Bergmann et al. use whole-tissue stretching and mathematical modeling to dissect the roles of mechanical stress and cell shape in cell division. They show that division orientation in stretched tissue is regulated indirectly by changes in cell shape, while division rate is more directly regulated by mechanical stress.

Nestor-Bergmann et al., 2019, Cell Reports *26*, 2088–2100
February 19, 2019 © 2019 The Authors.
https://doi.org/10.1016/j.celrep.2019.01.102

OPEN ACCESS
CellPress

Cell Reports
Article

# Decoupling the Roles of Cell Shape and Mechanical Stress in Orienting and Cueing Epithelial Mitosis

Alexander Nestor-Bergmann,[1,2,3,5,*] Georgina A. Stooke-Vaughan,[1,4,5] Georgina K. Goddard,[1,5] Tobias Starborg,[1] Oliver E. Jensen,[2] and Sarah Woolner[1,6,*]
[1]Wellcome Trust Centre for Cell-Matrix Research, Division of Cell Matrix Biology and Regenerative Medicine, School of Biological Sciences, Faculty of Biology, Medicine & Health, Manchester Academic Health Science Centre, University of Manchester, Oxford Road, Manchester, M13 9PT, UK
[2]School of Mathematics, University of Manchester, Manchester, M13 9PL, UK
[3]Present address: Department of Physiology, Development and Neuroscience, University of Cambridge, Cambridge, CB2 3DY, UK
[4]Present address: Department of Mechanical Engineering, University of California, Santa Barbara, Santa Barbara, CA, USA
[5]These authors contributed equally
[6]Lead Contact
*Correspondence: an529@cam.ac.uk (A.N.-B.), sarah.woolner@manchester.ac.uk (S.W.)
https://doi.org/10.1016/j.celrep.2019.01.102

## SUMMARY

**Distinct mechanisms involving cell shape and mechanical force are known to influence the rate and orientation of division in cultured cells. However, uncoupling the impact of shape and force in tissues remains challenging. Combining stretching of *Xenopus* tissue with mathematical methods of inferring relative mechanical stress, we find separate roles for cell shape and mechanical stress in orienting and cueing division. We demonstrate that division orientation is best predicted by an axis of cell shape defined by the position of tricellular junctions (TCJs), which align with local cell stress rather than tissue-level stress. The alignment of division to cell shape requires functional cadherin and the localization of the spindle orientation protein, LGN, to TCJs but is not sensitive to relative cell stress magnitude. In contrast, proliferation rate is more directly regulated by mechanical stress, being correlated with relative isotropic stress and decoupled from cell shape when myosin II is depleted.**

## INTRODUCTION

Cell division orientation and timing must be carefully regulated in order to shape tissues and determine cell fate, preventing defective embryonic development and diseases such as cancer (Mishra and Chan, 2014; Pease and Tirnauer, 2011; Quyn et al., 2010). Recent work has shown that mechanical cues from the extracellular environment can influence cell division rate (Benham-Pyle et al., 2015; Streichan et al., 2014) and orientation (Campinho et al., 2013; Finegan et al., 2019; Legoff et al., 2013; Mao et al., 2011; Fink et al., 2011). What remains unclear is whether dividing cells are directly sensing mechanical forces or are responding to changes in cell shape induced by these forces. This distinction is crucial because the molecular mechanisms involved in either shape or force sensing could be very different (Luo et al., 2013; Nestor-Bergmann et al., 2014).

Several mechanisms of division orientation control have been postulated in single cells, with evidence for both shape and stress sensing (Fink et al., 2011; Minc et al., 2011; Minc and Piel, 2012; Théry et al., 2006). There is limited understanding of how these models could apply to tissues, where cells are linked together by adhesions and it is far more difficult to exclusively manipulate either cell shape or mechanical stress. Recent evidence for a shape-sensing mechanism was found in the *Drosophila* pupal notum. The spindle orientation protein Mud (*Drosophila* ortholog of NuMA) localizes at tricellular junctions (TCJs), recruiting force generators to orient astral microtubules in rounding mitotic cells (Bosveld et al., 2016). However, this mechanism has yet to be demonstrated in another system or related to mechanical stress. In contrast, recent work in a stretched monolayer of MDCK cells has indicated that division orientation may be mediated by a tension-sensing mechanism requiring E-cadherin, although an additional role for cell shape sensing could not be excluded (Hart et al., 2017). Indeed, divisions in MDCK cells have also been found to align better with cell shape than a global stretch axis, though local cell stress was not known in this case (Wyatt et al., 2015).

Separating the roles of shape and stress in tissues will inevitably require an understanding of how force is distributed through heterogeneous cell layers. Experimental methods of assessing stress include laser ablation, atomic force microscopy, and micro-aspiration (Campinho et al., 2013; Davidson et al., 2009; Hoh and Schoenenberger, 1994; Hutson et al., 2003). While informative, these techniques are invasive, perturbing the stress field through the measurement, and usually require constitutive modeling for the measurement to be interpreted (Stooke-Vaughan et al., 2017; Sugimura et al., 2016). However, mathematical modeling combined with high-quality


This is an open access article under the CC BY license (http://creativecommons.org/licenses/by/4.0/).

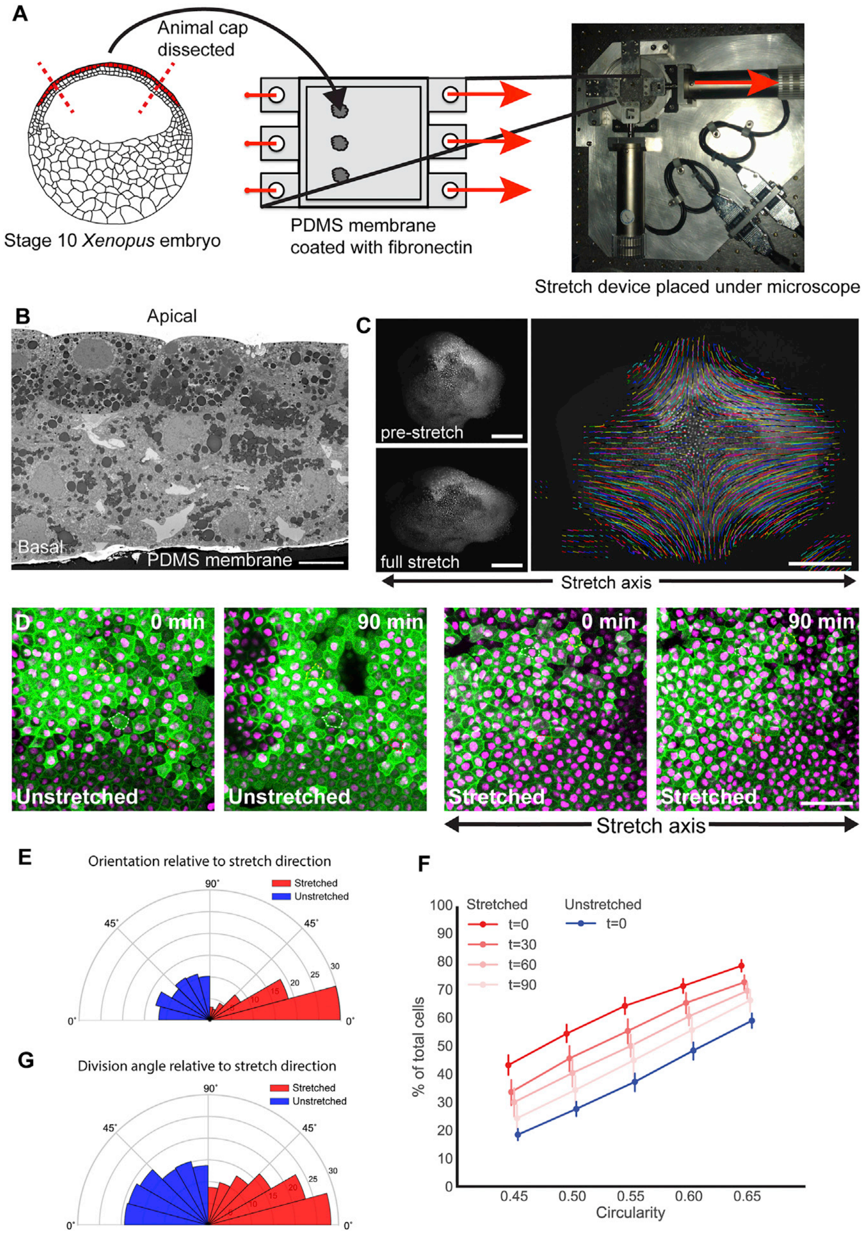


**Figure 1. Application of Tensile Force to a Multi-layered Tissue**

(A) Animal cap tissue was dissected from stage 10 *Xenopus laevis* embryos and adhered to fibronectin-coated PDMS membranes, and a 35% uniaxial stretch of the membrane was applied.

(B) 3View scanning electron micrograph showing that the cultured animal cap tissue is two to three cells thick. Cell shape and divisions were assessed in the apical cell layer.

(C) Displacement of nuclei was tracked in a stretched animal cap.

(D) Confocal images of the apical cells in unstretched and stretched animal caps (green, GFP-alpha-tubulin; magenta, cherry-histone2B), taken 0 and 90 min after stretch. Representative cells outlined by dashed lines.

(E) Rose plot showing orientation of cell shape relative to direction of stretch in unstretched (blue) and stretched (red; measured immediately following stretch) experiments.

(F) Cumulative plots of cell circularity in unstretched (blue) and stretched (red; at 0, 30, 60 and 90 min after stretch) animal caps (0 = straight line, 1 = circle). One hundred percent of cells have circularity $\leq 1$. Markers are slightly offset for clarity. Error bars represent 95% confidence intervals.

(G) Rose plot of division angle relative to direction of stretch for unstretched (blue) and stretched (red) experiments. Kolmogorov-Smirnov test indicates that the unstretched distribution is not significantly different from a uniform distribution, n = 343 divisions, 15 animal caps; Kolmogorov-Smirnov test indicates that stretched distribution is significantly different from uniform, $p < 1.4 \times 10^{-9}$, n = 552 divisions, 17 animal caps.

Scale bars, 10 μm in (B), 500 μm in (C), and 50 μm in (D).

fluorescence imaging now provides the possibility of non-invasively inferring mechanical stress in tissues (Brodland et al., 2014; Chiou et al., 2012; Feroze et al., 2015; Ishihara and Sugimura, 2012; Nestor-Bergmann et al., 2018a; Xu et al., 2015).

In this work, we apply a reproducible strain to embryonic *Xenopus laevis* tissue to investigate the roles of shape and stress in cell division in a multi-layered tissue. We particularly focus on mathematically characterizing local (cell-level) and global (tissue-level) stress and the relation to cell shape and division. Our data suggest that mechanical stress is not directly sensed for orienting the mitotic spindle, acting only to deform cell shape, but is more actively read as a cue for mitosis.

## RESULTS

### Application of Tensile Force to a Multi-layered Embryonic Tissue

To investigate the relationship among force, cell shape, and cell division in a complex tissue, we developed a system to apply reproducible mechanical strain to a multi-layered embryonic tissue. Animal cap tissue was dissected from stage 10 *Xenopus laevis* embryos and cultured on a fibronectin-coated elastomeric poly-di-methyl-siloxane (PDMS) substrate (Figure 1A). A uniaxial stretch was applied to the PDMS substrate using an automated stretch device (Figure 1A) and imaged using standard microscopy. The three-dimensional structure of the stretched tissue (assessed using 3View EM) could be seen to comprise approximately three cell layers (Figure 1B), as would be expected in a stage 10 *Xenopus laevis* embryo (Keller, 1980; Keller and Schoenwolf, 1977), therefore maintaining the multi-layered tissue structure present *in vivo*.

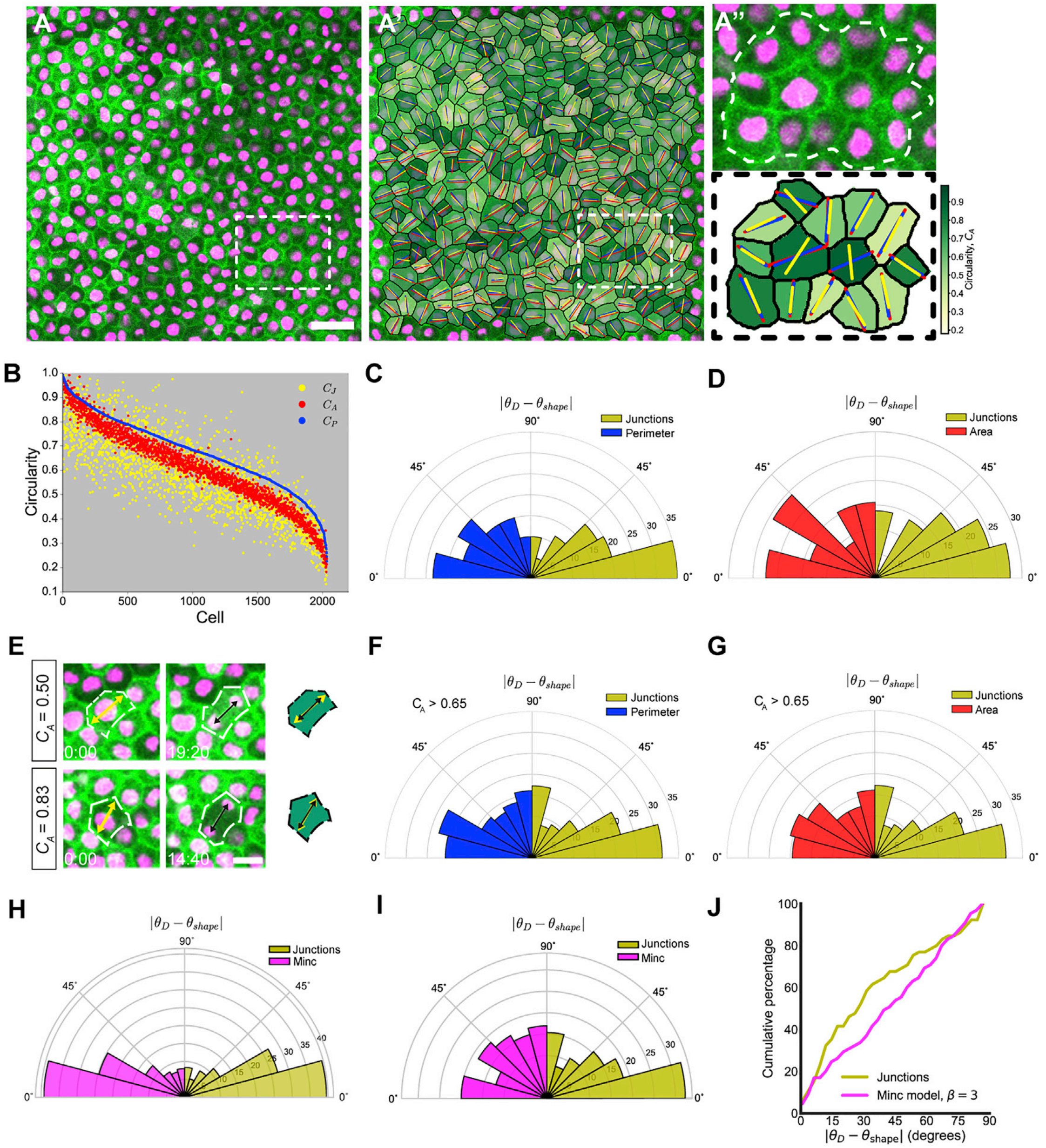


**Figure 2. Cell Division Orientation Is Best Predicted by an Axis of Shape Defined by TCJs**

(A) Representative image of control cells from an unstretched experiment. Scale bar, 20 μm.

(A′) Overlay of segmentation of cells given in (A), with the principal axis of shape characterized by area, perimeter, and junctions drawn in red, blue, and yellow, respectively.

(A″) Enlargement of segmented cells from white box drawn in (A′); cells analyzed are outlined by dashed white line.

(B) Circularities of 2,035 cells from unstretched experiments, with shape characterized by area, perimeter, and junctions plotted in red, blue, and yellow respectively. Cells have been ordered in descending order of perimeter-based circularity ($C_P$), with the corresponding values of $C_A$ and $C_J$ plotted alongside.

(C) Rose plot of difference between division angle, $\theta_D$, and orientation of shape on the basis of perimeter (blue; $\theta_{shape} = \theta_P$) and junctions (yellow; $\theta_{shape} = \theta_J$), for cells that satisfy $|\theta_P - \theta_J| \geq 15^\circ$.

(D) Rose plot of difference between division angle, $\theta_D$, and orientation of shape on the basis of area (red; $\theta_{shape} = \theta_A$) and junctions (yellow; $\theta_{shape} = \theta_J$), for cells that satisfy $|\theta_A - \theta_J| \geq 15^\circ$.

(E) Examples of elongated (top) and round (bottom) cells where division angle (black arrows) is well predicted by the principal axis of shape defined by area (yellow arrows).

(F) Rose plot of difference between division angle, $\theta_D$, and orientation of shape on the basis of perimeter (blue; $\theta_{shape} = \theta_P$) and junctions (yellow; $\theta_{shape} = \theta_J$), for round cells that satisfy $C_A > 0.65$.

*(legend continued on next page)*

## Stretching Elongates Cell Shape and Reorients Divisions

A 35% stretch of the PDMS substrate led to a 19.67 ± 1.91% (95% confidence interval) elongation of the most apical cells in the animal cap (also known as the superficial layer) along the stretch axis (measured change in length of one-dimensional lines drawn on opposite sides of the animal cap; displacement field shown in Figure 1C). The difference in elongation between the substrate and apical cells is presumably a result of the mechanical stress being dissipated through multiple cell layers. The qualitative change in cell shape was not as substantial as was previously observed in stretched monolayers (Wyatt et al., 2015) (Figure 1D).

We mathematically characterized shape using two parameters: orientation of the principal axis of cell shape relative to the stretch axis (0°), $\theta_A$, and cell circularity, $C_A$ (derived in Section 1.4 of Methods S1 in the Supplemental Information). $C_A$ describes the degree of elongation of a cell (ranging from 0 being a straight line to 1 being a perfect circle), and $\theta_A$ indicates the principal direction in which this occurs. Stretching oriented the majority of cells with the direction of stretch (Figure 1E) and caused a highly reproducible elongation of cell shape (Figure 1F). However, when the substrate was held fixed following stretch, cell elongation reduced over time and returned close to the unstretched shape profile after 90 min (95% confidence intervals of stretched animal caps at t = 90 min overlap with unstretched caps; Figure 1F). Therefore, cells in this tissue adapt to the elongation caused by stretching and do not behave like a purely elastic material.

In unstretched tissue, division orientation, $\theta_D$, was not significantly different from a uniform distribution (p = 0.36, Kolmogorov-Smirnov test; Figure 1G). In contrast, divisions in the stretched tissue were significantly oriented along the axis of stretch, ($p < 1.43 \times 10^{-9}$, Kolmogorov-Smirnov test; Figure 1G), with 52% of divisions oriented within 30° of the stretch axis (compared with 36% in unstretched).

## Shape-Based Models of Division Differ Significantly Depending on the Cellular Characteristics Used to Define Shape

A shape-based "long-axis" division rule may explain why stretching reorients divisions. However, the precise molecular mechanism behind shape-based models remains unclear and may vary across cell type and tissue context (Campinho et al., 2013; Fink et al., 2011; Minc et al., 2011). Past models have used different characteristics to determine the shape of a cell, usually selecting one of the following: cell area, cell perimeter, and TCJ (which we define here as the meeting point of three or more cells). Although often used interchangeably, these shape characteristics model different biological functions. We investigated their differences and determined if one characteristic predicts division orientation better than the others.

We modeled cell shape by area, perimeter, and TCJs to derive three respective measures of cell shape orientation, $\theta_A$, $\theta_P$, and $\theta_J$, and circularity, $C_A$, $C_P$, and $C_J$ (Methods S1, Section 1). Cells tend to have $C_P > C_A > C_J$ (i.e., shape generally appears less anisotropic using the perimeter-based measure). $C_A$ and $C_P$ (and correspondingly $\theta_A$ and $\theta_P$) are reasonably well correlated, whereas $C_J$ (and $\theta_J$) tends to coincide less well with the others (Figures 2A, 2B, and S1A). Thus a cell that appears round by area and perimeter can have clear elongation as measured by TCJs. This is intuitive for rounding mitotic cells, where TCJs can be distributed non-uniformly around the circular periphery (Bosveld et al., 2016). However, it is surprising that this can also be the case in cells with relatively straight edges (Figure 2A″; note how $\theta_J$ [yellow line] differs from $\theta_A$ and $\theta_P$ [blue and red lines] in the central dark green cells). Notably, cells in the *Xenopus* animal cap do not undergo the dramatic mitotic cell rounding seen in some other systems (Bosveld et al., 2016) (Figures S1B and S1C).

## TCJ Placement Is a Better Predictor of Division Orientation Than Cell Area, Cell Perimeter, or Microtubule Length

Given that $\theta_A$, $\theta_P$, and $\theta_J$ are often highly correlated, division orientation is generally well predicted by all three. We therefore focused on cases in which the orientations of shape differed by at least 15°. In a pooled sample of 600 cells from stretched and unstretched tissue, only 7 cells were found to have $|\theta_A - \theta_P| \geq 15^\circ$. Fifty-eight cells satisfied $|\theta_A - \theta_J| \geq 15^\circ$, and 60 satisfied $|\theta_P - \theta_J| \geq 15^\circ$. In the latter two cases, $\theta_J$ was a significantly better predictor of division angle than random ($p < 0.0162$ when $|\theta_A - \theta_J| \geq 15^\circ$ and $p < 0.0042$ when $|\theta_P - \theta_J| \geq 15^\circ$, Mann-Whitney U test), but $\theta_A$ and $\theta_P$were not (Figures 2C and 2D). Furthermore, $C_A$, $C_P$, and $C_J$ were all significantly higher in these subpopulations (Figures S1D and S1E; 95% confidence intervals do not overlap), indicating that these cells are rounder yet can still effectively orient their spindles in line with their TCJs. This result is strengthened considering that TCJs provide fewer data points than area or perimeter, so junctional data may be more susceptible to geometric error than area and perimeter. For all of our data comparing cell shape with division orientation, we use shape determined just prior to nuclear envelope breakdown (NEB), avoiding any possible shape changes due to mitosis (e.g., cell rounding on entry into mitosis or elongation at anaphase). However, to test whether the fidelity of division alignment to TCJ shape changes depending on when shape is measured, we compared $|\theta_D - \theta_J|$ at time points through mitosis, finding no significant difference (Figure S1F). It is important to note that we do not see significant

(G) Rose plot of difference between division angle, $\theta_D$, and orientation of shape on the basis of area (red; $\theta_{shape} = \theta_A$) and junctions (yellow; $\theta_{shape} = \theta_J$), for round cells that satisfy $C_A > 0.65$. See also Figure S1.

(H) Rose plot of difference between division angle, $\theta_D$, and orientation of shape on the basis of Minc model when $\beta = 3$ (magenta; $\theta_{shape} = \theta_{Minc}$) and junctions (yellow; $\theta_{shape} = \theta_J$) for all cells in stretched and unstretched experiments (n = 599 cells).

(I) Rose plot of difference between division angle, $\theta_D$, and orientation of shape on the basis of Minc model when $\beta = 3$ (magenta; $\theta_{shape} = \theta_{Minc}$) and junctions (yellow; $\theta_{shape} = \theta_J$), for cells that satisfy $|\theta_{Minc} - \theta_J| \geq 15^\circ$ (n = 65 cells).

(J) Cumulative plot of difference between division angle, $\theta_D$, and orientation of shape for data shown in (I).

OPEN
ACCESS
CellPress

cell rounding in the *Xenopus* animal cap upon entry into mitosis (Figure S1C), so static fidelity is likely a reflection of relatively static cell shape in this system, a feature which helps simplify our analysis.

In unstretched tissue, cells that we classed as "rounded" ($C_A >$ 0.65; Figure 2E) showed no significant correlation between $\theta_A$ and $\theta_D$ or $\theta_P$ and $\theta_D$, as could be expected from previous work (Minc et al., 2011). However, $\theta_J$ was significantly aligned with division angle in these round cells compared with random ($p = 0.025$, Mann-Whitney U test) (Figures 2F and 2G). This degree of sensitivity is striking and further demonstrates that TCJ sensing could function effectively in round cells, which may have previously been thought to divide at random. Our analysis is based purely on predictions arising from the data and thereby has the advantage of being independent of unknown model parameters and assumptions. However, to test how our division predictions compare with previous models of division orientation, we turned to a well-known shape-based model of division in isolated cells (Minc et al., 2011). The "Minc" model hypothesizes that astral microtubules exert length-dependent pulling forces on the spindle, thereby exerting a torque and rotating the spindle, with division predicted to occur along the axis of minimum torque. In this shape-based model, the shape of the cell determines the distribution of torque on the spindle and thereby the division axis (see Methods S1 for further details of this model and its implementation). As with our purely geometric measures of shape, we found that the Minc model predicts division orientation significantly better than a random distribution (Figure 2H; $p < 4.1 \times 10^{-40}$ for TCJs and $p < 1.2 \times 10^{-39}$, Mann-Whitney U test). However, for cells where the predicted division axes according to TCJs ($\theta_J$) and the Minc model ($\theta_{Minc}$) differed by more than 15°, TCJs ($\theta_J$) provided a prediction of division angle that was significantly better than random ($p < 0.028$, Mann-Whitney U test), whereas division predicted by microtubule pulling forces ($\theta_{Minc}$) did not (Figures 2I and 2J), indicating that TCJs provide a better prediction of division orientation. This result held for multiple scaling laws between microtubule length and force (Figures S1G and S1H).

## Local Cell Shape Aligns with Local Stress and Predicts Division Orientation Better Than Global Stretch and Stress

Contrary to observations in monolayers (Hart et al., 2017), we found that cells in stretched tissue divide according to cell shape both when $\theta_J$ is oriented with (Figure 3A) and against (Figures 3B and 3C) the direction of stretch. Moreover, in the case of cells that are relatively round in shape ($C_J > 0.65$), there is no preference for aligning with the global stretch direction, and indeed alignment with TCJ shape still appears more accurate than with the stretch axis (Figures S2A and S2B; $p < 0.005$ for TCJs, not significant for stretch direction, Mann-Whitney U test). These data indicate that global stretch direction is a poor predictor of division angle compared with cell shape. However, little is known about the local stress distribution around individual cells in a tissue subjected to a stretch, which may not coincide with global stress in such a geometrically heterogeneous material.

We extended a popular vertex-based model to mathematically characterize cell stress (Brodland et al., 2014; Chiou et al., 2012; Ishihara and Sugimura, 2012; Nestor-Bergmann et al., 2018a, 2018b). Predicted orientations of forces from the model have been found to be in accordance with laser ablation experiments (Farhadifar et al., 2007; Landsberg et al., 2009), indicating that the model can provide a physically relevant description of cellular stresses. Our methodology allows relative cell stress to be inferred solely from the positions of cell vertices, without invasively altering the mechanical environment (Methods S1, Section 2). The model predicts that the orientation of cell shape based on TCJs, $\theta_J$, aligns exactly with the principal axis of local stress (Nestor-Bergmann et al., 2018a) (Figure 3D). We demonstrated this computationally in stretched tissue by simulating a uniaxial stretch (Figures 3E and 3F). Following stretch, we see that local cell stress remains aligned with $\theta_J$, rather than the global stress along the x axis. Much previous work assumes that the local axis of stress coincides with the global stress. Significantly, the model predicts that a stress-sensing mechanism would align divisions in the same direction as a shape-based mechanism (as in Figure 3B).

## The Magnitude of Cell Stress Does Not Correlate with the Alignment of Division Angle and TCJ Positioning

If a stress-sensing mechanism were contributing to orienting division, we hypothesized that cells under higher net tension or compression might orient division more accurately with the principal axis of stress ($\theta_J$). We infer relative tension and compression using the isotropic component of stress, effective pressure ($P^{\text{eff}}$) (Nestor-Bergmann et al., 2018a):

$$P^{\text{eff}} = \frac{\tilde{A}}{\tilde{A}_0} - 1 + \frac{\Gamma \tilde{L}^2}{2\tilde{A}} + \frac{\Lambda \tilde{L} \sqrt{\tilde{A}_0}}{4\tilde{A}},$$

where $\tilde{A}$ is cell area, $\tilde{L}$ is perimeter, $\tilde{A}_0$ is the preferred area, and $(\Lambda, \Gamma)$ are model parameters, defined in Section 2 of Methods S1 and inferred from data (Video S1) (Nestor-Bergmann et al., 2018a). Cells under net tension have $P^{\text{eff}} > 0$, whereas $P^{\text{eff}} < 0$ indicates net compression. We provide a mathematical method for estimating $\tilde{A}_0$ in Section 3 of Methods S1. A representative segmentation, showing cells predicted to be under net tension and compression, from an unstretched experiment is given in Figure 3G. Interestingly, we found no correlation between the value of $P^{\text{eff}}$ (relative isotropic stress) and the alignment of division orientation to $\theta_J$ ($|\theta_D - \theta_J|$) (Figure S2C). The mechanical state of a cell may also be characterized by shear stress, $\xi$ (defined as the eigenvalue of the deviatoric component of the stress tensor; see Section 2 of Methods S1). Larger values of $|\xi|$ indicate increased cellular shear stress. Again, we found no correlation between $\xi$ and the alignment of division to $\theta_J$ (Figure S2D).

Despite the lack of correlation with stress magnitude, cell shape anisotropy, measured by $C_J$, correlates significantly with $|\theta_D - \theta_J|$ ($p < 3.04 \times 10^{-10}$, Spearman rank correlation coefficient; Figure 3H), with elongated cells having $\theta_D$ aligned with $\theta_J$ significantly better than round cells ($p < 1.64 \times 10^{-8}$; Figure 3I).

## Cadherin Is Required for Positioning the Mitotic Spindle Relative to Cell Shape

Immunofluorescence staining of β-catenin confirmed that adherens junctions were distributed along the apical cell cortex but

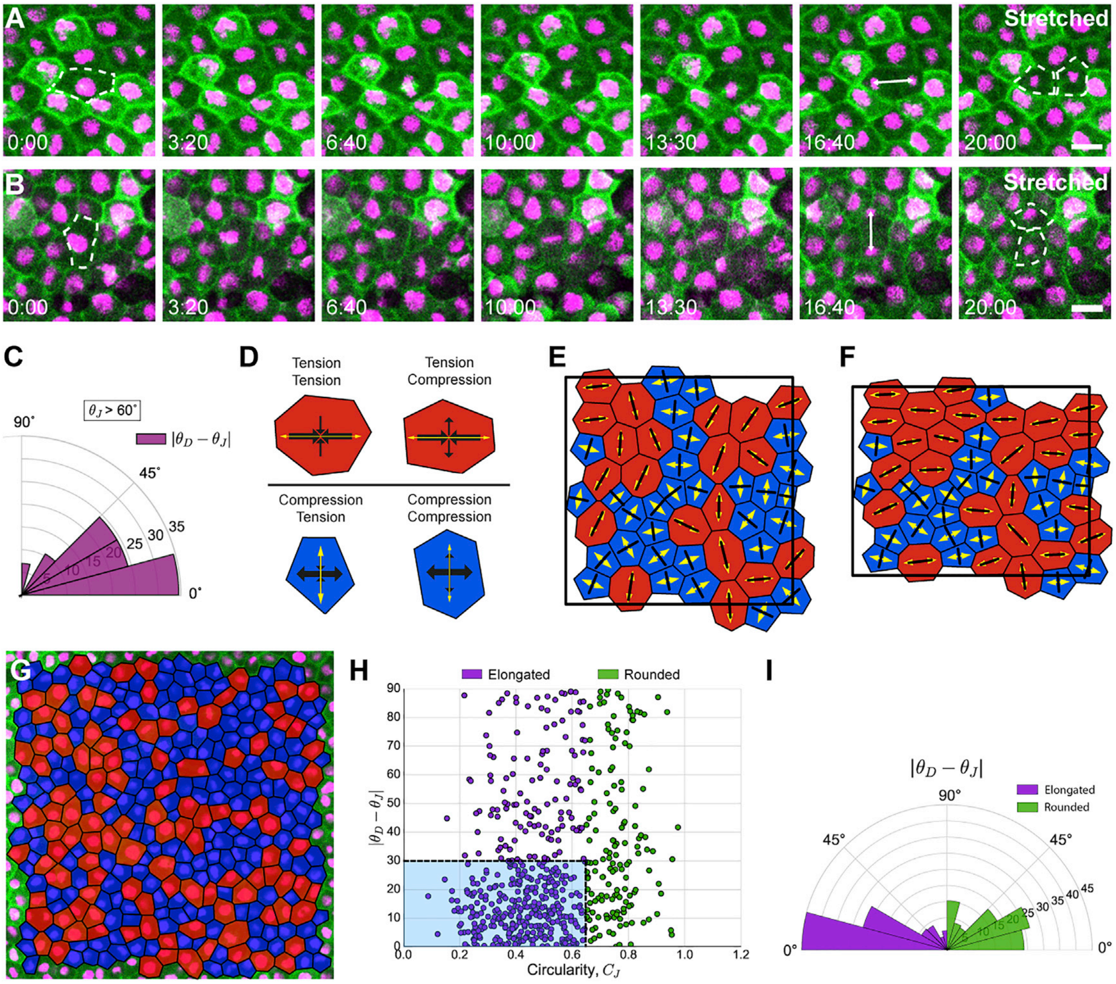


**Figure 3. Division Orientation Is Better Predicted by Shape Rather Than High Relative Isotropic or Shear Stress**

(A) Images taken from a confocal time-lapse video of a division in a cell in stretched tissue whose interphase shape (dashed line, 0:00) is oriented with the stretch (horizontal) axis. Cell division aligns with both cell shape and stretch axis.

(B) Time-lapse images of an unusual cell in a stretched tissue, whose interphase shape (dashed line, 0:00) is oriented against the stretch axis. Cell division aligns with cell shape but against the stretch axis.

(C) Rose plot of difference between division angle, $\theta_D$, and orientation of shape on the basis of junctions, $\theta_J$, for cells from stretched experiments, where $\theta_J$ was at least 60° divergent to the direction of stretch. Twenty-nine cells satisfied this condition. Kolmogorov-Smirnov test found a significant difference from a uniform distribution (p = 0.022).

(D) Representative cells showing classification of cell stress configurations. Red (blue) cells are under net tension (compression), where $P^{eff}$ is positive (negative). Larger (smaller) black arrows indicate the orientation of the principal (secondary) axis of stress, with inward- (outward)-pointing arrows indicating the tension (compression) generated by the cell. Yellow arrows indicate the principal axis of shape defined by cell junctions, which aligns exactly with a principal axis of stress.

(E) Fifty simulated cells randomly generated in a periodic box, relaxed to equilibrium with parameters $(\Lambda,\Gamma) = (-0.259, 0.172)$, under conditions of zero global stress (Nestor-Bergmann et al., 2018a). Red (blue) cells are under net tension (compression). Principal axis of stress (shape) indicated in black (yellow).

(F) Cells from (E) following a 13% area-preserving uniaxial stretch along the x axis.

(G) Example segmented cells from an unstretched experiment. Cells in red (blue) are predicted to be under net tension (compression).

(H) Cell circularity defined by junctions, $C_J$, versus $|\theta_D - \theta_J|$. Spearman rank correlation coefficient found a significant correlation ($p < 3.04 \times 10^{-10}$). Elongated cells ($C_J \leq 0.65$) cluster in blue box, whereas rounded cells ($C_J > 0.65$) have a more uniform distribution.

(I) Rose plot of difference between division angle, $\theta_D$, and orientation of shape on the basis of junctions, $\theta_J$ for round ($C_J > 0.65$; right) and elongated ($C_J \leq 0.65$; left) cells shown in (H). Mann-Whitney U test indicated that elongated cells have $\theta_J$ aligned significantly more with $\theta_D$ than rounded cells ($p < 1.64 \times 10^{-8}$).

Scale bars in (A) and (B), 20 μm. All rose plots show percentage of cells.

See also Figure S2.

particularly concentrated at the meeting points of three or more cells (Figure 4A). To test a functional requirement for adherens junctions in orienting the spindle, we focused on maternal C-cadherin (cadherin 3), which is expressed at the highest level in stage 10 and 11 *Xenopus* embryos (Heasman et al., 1994; Lee and Gumbiner, 1995). We used two constructs to manipulate

OPEN ACCESS
CellPress

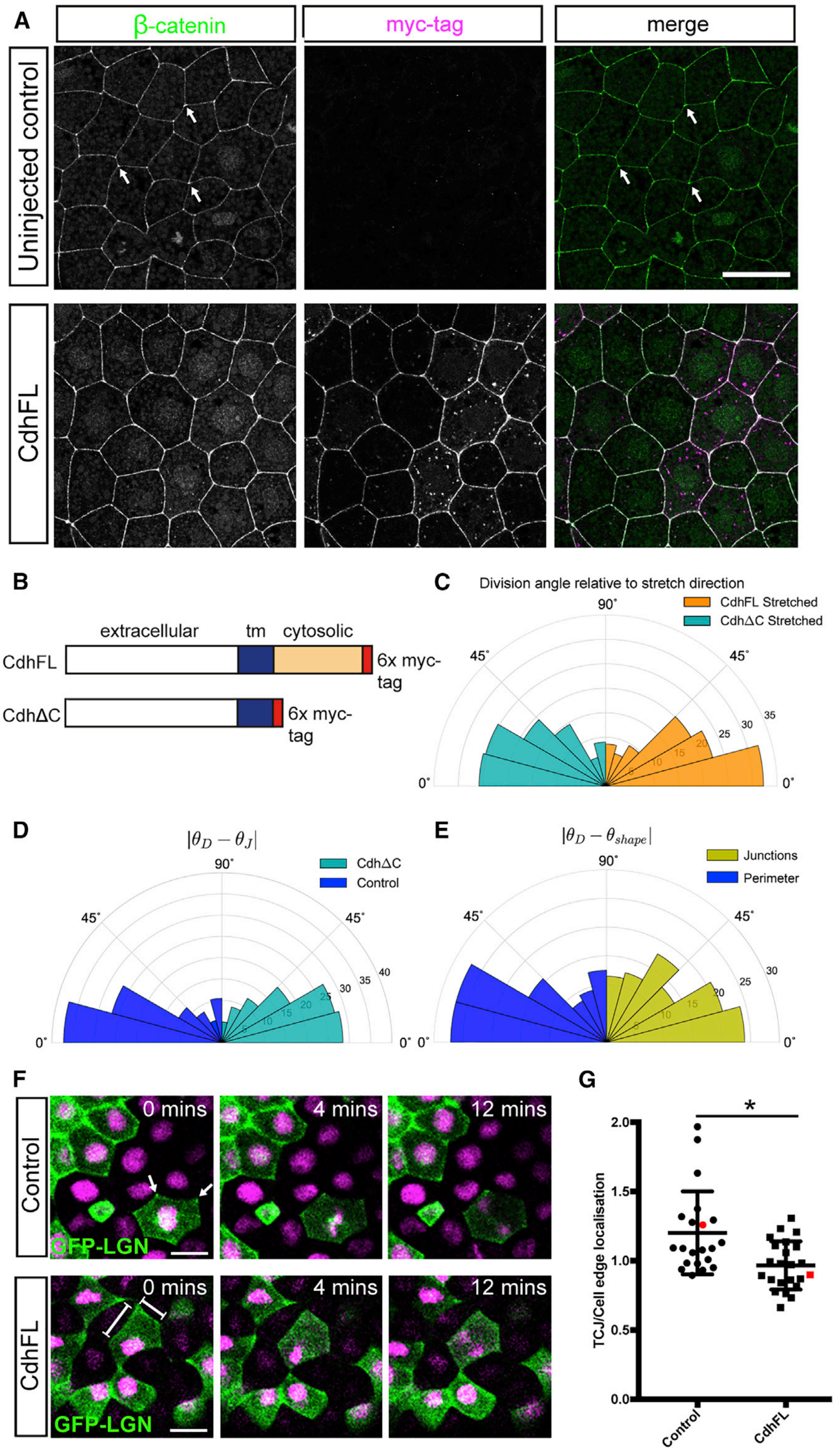


**Figure 4. C-Cadherin Is Required to Orient the Mitotic Spindle According to Cell Shape**

(A) Single confocal slices from immunofluorescent staining for β-catenin (green) and myc-tag (magenta) in uninjected and CdhFL-injected stage 12 embryos (stage matched to time that animal caps are stretched and imaged). Hotspots of β-catenin localization (arrows) are seen at TCJs in controls but are lost when CdhFL is overexpressed.

(B) Schematic of Cadherin constructs CdhFL and CdhΔC.

(C) Rose plot of division angles, $\theta_D$, relative to direction of stretch for cells from stretched CdhΔC-injected (411 cells; cyan) and stretched CdhFL-injected experiments (552 cells; orange). CdhFL-injected cells align significantly better with direction of stretch ($p < 0.0162$, Mann-Whitney U test).

(D) Rose plot of difference between division angle, $\theta_D$, and orientation of shape on the basis of junctions, $\theta_J$, for cells from CdhΔC-injected experiments (390 cells; cyan) and control experiments (239 cells; blue). Distributions are significantly different ($p < 0.016$ Kolmogorov-Smirnov test).

(E) Rose plot of difference between division angle, $\theta_D$, and orientation of shape on the basis of perimeter, $\theta_P$, (blue) and junctions, $\theta_J$, (yellow) for 96 cells from CdhFL-injected experiments that satisfied $|\theta_P - \theta_J| \geq 15^{\circ}$. $\theta_D$ aligns significantly better to $\theta_P$ than a random distribution ($p < 0.004$; Kolmogorov-Smirnov test), but not to $\theta_J$.

(F) Images from time-lapse videos of control and CdhFL-injected animal cap tissue expressing GFP-LGN in a mosaic fashion. In control cells, GFP-LGN is enriched at TCJs during interphase (arrows), and this localization persists through mitosis. The enrichment of GFP-LGN at TCJs is lost when CdhFL is expressed, with localization spread throughout the cell edge (line).

(G) Quantification of GFP-LGN localization at TCJs compared with cell edges in single mitotic cells in animal caps. GFP-LGN is more strongly localized at TCJs compared with cells edges in controls, but this bias is lost in CdhFL-injected tissue (*$p < 0.05$, Kolmogorov-Smirnov test; n = 21 and 23 mitotic cells from seven and six unrelated animal caps for control and CdhFL, respectively). Error bars represent mean and SD.

Red points show quantification for mitotic cells highlighted in (F). Rose plots show percentage of cells. Scale bars, 20 μm.

See also Figure S3.

C-cadherin in the tissue: C-cadherin FL -6xmyc (CdhFL; full-length C-cadherin with 6xmyc tags at the intracellular C terminus) and C-cadherin ΔC -6xmyc (CdhΔC; C-cadherin with extracellular and transmembrane domains but lacking the cytosolic domain) (Figure 4B) (Kurth et al., 1999). CdhFL- and CdhΔC-injected embryos developed normally up to stage 10 or 11 (Figure S3A), but the majority of embryos failed to complete gastrulation (Lee and Gumbiner, 1995, and data not shown). We observed no change in the cumulative distribution of cell circularities in CdhFL- and CdhΔC-injected tissues compared with control tissue (Figure S3B). We also saw no difference in the rate of cell divisions (data not shown).

CdhΔC-injected tissue was elongated by application of stretch (Figure S3C) but showed a worse alignment of divisions to stretch direction compared to uninjected control and CdhFL-injected tissue (Figure 4C; $p < 0.0162$ for CdhΔC less than CdhFL, Mann-Whitney U test). Moreover, unstretched CdhΔC-injected tissue showed a significant decrease in the alignment of division angle to $\theta_J$ compared with uninjected controls (Figure 4D; $p < 0.016$, Kolmogorov-Smirnov test on distributions differing), though both were significantly different to random (control, $p < 3.6 \times 10^{-11}$; CdhΔC, $p < 4.3 \times 10^{-11}$; Kolmogorov Smirnov test). To further investigate a requirement for adherens junctions in division orientation, we overexpressed C-cadherin in the cell cortex by injecting CdhFL. Focusing on cells that satisfied $|\theta_P - \theta_J| \geq 15^\circ$, we found the striking result that division orientation was now significantly well predicted by cell perimeter but no longer by TCJs (Figure 4E; $p < 0.0027$ for alignment $\theta_D$ to $\theta_P$ but not significant for $\theta_D$ to $\theta_J$, Mann-Whitney U test). Therefore, overexpression of CdhFL was sufficient to switch division orientation from alignment with TCJs to alignment with the shape of the entire cortex.

To investigate the mechanism behind the observed switch in division orientation, we explored how overexpression of CdhFL alters the localization of spindle orientation machinery at the cell cortex. We found that overexpression of CdhFL led to a loss of the ''hotspots'' of β-catenin localization at TCJs seen in control tissue, in both interphase and mitotic cells (Figures 4A and S3D). When CdhFL is overexpressed, β-catenin is more equally spread around the entire apical perimeter of the cell (Figures 4A, S3D, and S3E). The ''hotspots'' of β-catenin localization in controls are not purely a result of more cells' contributing to this focal point but are also seen when fluorescence intensity is measured in single β-catenin-GFP-expressing cells in the animal cap (Figures S3D and S3E). To determine how this observed change in adherens junction localization might alter spindle orientation, we investigated how the localization of the spindle orientation protein, LGN, was altered by overexpression of CdhFL. Mosaic expression of GFP-LGN allowed us to analyze at the single cell level in stretched and unstretched animal caps. In control tissue, LGN, like β-catenin, shows a more concentrated localization at TCJs (Figures 4F and 4G). We observed no significant difference in LGN localization between unstretched and stretched tissue (data not shown). However, we saw a loss of concentrated ''hotspots'' of LGN localization when CdhFL is overexpressed, with LGN instead spread more equally around the whole perimeter (Figures 4F and 4G). We therefore suggest that overexpression of CdhFL switches division orientation from alignment with TCJs to alignment with the shape of the whole cortex by altering the localization of LGN.

## Cell Division Rate Is Temporarily Increased following Change in Global Stress

Stretch elicited a reproducible and significant increase in cell division rate, with 6.47 ± 1.12% of cells dividing per hour in the stretched tissue compared with 3.22 ± 0.55% in unstretched tissue (Figure 5A; 95% confidence intervals do not overlap), as reported for cultured cells and monolayers (Fink et al., 2011; Streichan et al., 2014; Wyatt et al., 2015). We roughly classify two distinct periods of division after stretch; there is an initial period of high proliferation (8.1% of cells undergoing division per hour; Figure 5B), which drops, after 40–60 min, to near unstretched control levels (4.2% of cells undergoing division per hour). Stretching increases apical tissue area by 6 ± 2.69% (95% confidence interval) and is predicted to increase global stress by increasing individual values of $P^{\mathrm{eff}}$. We sought to determine whether the increase in division rate is a response to these changes.

In both stretched and unstretched experiments, dividing cells had a larger area than the population, being about 22.7% and 25.7% larger on average respectively (Figure 5C). Similarly, the mean perimeter was significantly larger in the dividing cells by about 14.1% in unstretched and 13.8% in stretched (Figure 5D). However, there was no significant difference in the level of cell elongation in dividing cells (Figure S2E). Crucially, we found that dividing cells were more likely to be under predicted net tension than compression (Figure 5E, more cells in red region). However, $P^{\mathrm{eff}}$ is correlated with cell area (though the two are not always equivalent), so a further perturbation was required to separate their effects.

## Loss of Myosin II Reduces Cell Contractility

We perturbed the mechanical properties of the tissue with targeted knockdown of non-muscle myosin II using a previously published morpholino (Skoglund et al., 2008). As expected, myosin II knockdown disrupted cytokinesis, seen by the formation of ''butterfly''-shaped nuclei, where daughter cells had not fully separated (Figures 6A and 6B). However, division rate and orientation could still be assessed using the same methods described for control tissue. Myosin II is known to generate contractility within a tissue (Clark et al., 2014; Effler et al., 2006; Gutzman et al., 2015). Accordingly, we found evidence for reduced contractility in the myosin II morpholino (MO) tissue by observing that cells were much slower at adapting to stretch, remaining elongated for longer (compare Figure 6C with Figure 1F).

## Myosin II Is Required for Mitotic Entry in Unstretched Tissue

Somewhat surprisingly, considering suggestions that myosin II may play a stress-sensing role in orienting the spindle (Campinho et al., 2013), we found that alignment of division angle to stretch and $\theta_J$ was unaffected in global myosin II knockdown experiments (Figures 6D and 6E). In contrast, proliferation rate was significantly affected, with divisions virtually ceasing in unstretched myosin II MO tissue. Strikingly, stretching the myosin II MO tissue increased the division rate to significantly higher levels (Figure 6F). Thus myosin II is required to cue cells into division in the unstretched tissue, but this can be partially overridden by applying an external loading. Unlike in control experiments, dividing cells in myosin II knockdown stretch experiments were not significantly larger than the population in area (Figure 6G) or perimeter (Figure 6H), so cell area has been uncoupled as a cue to divide in the myosin II knockdowns. This finding, along with our observation that dividing cells were more likely to be under relative net tension than relative compression (Figure 5E), indicates that in a tissue the cue to

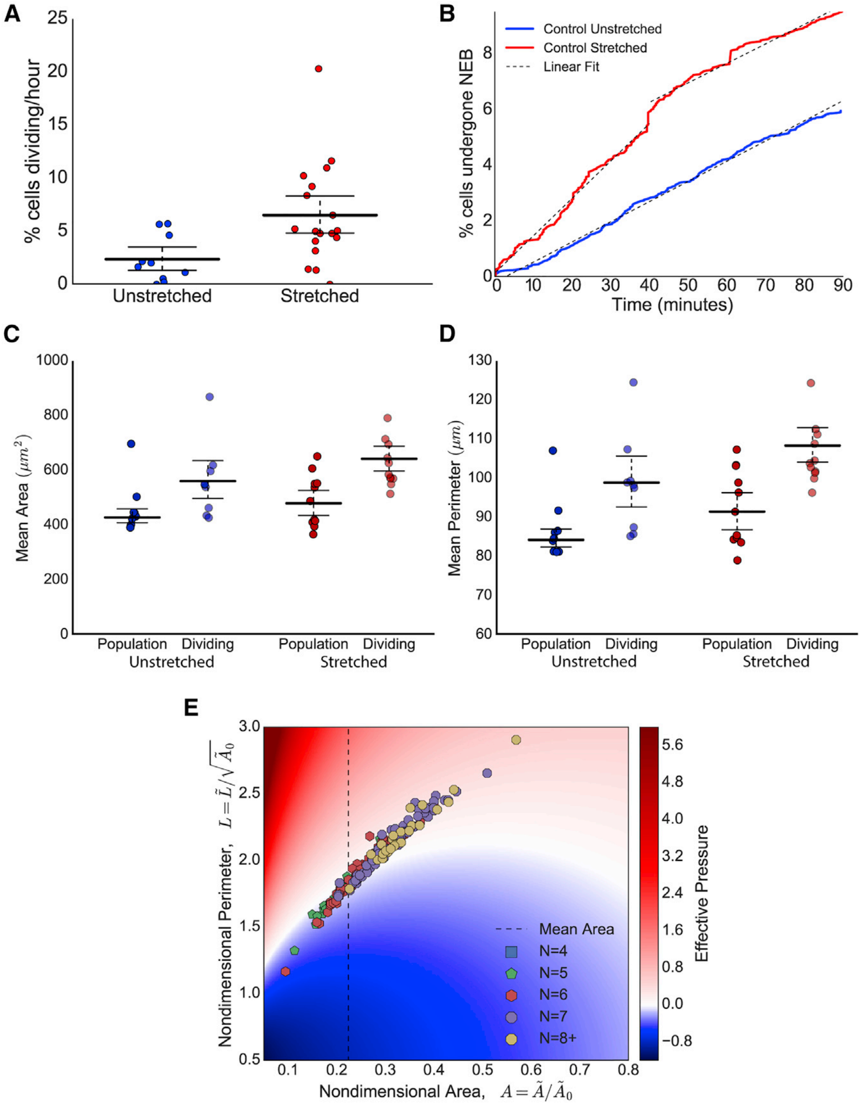


**Figure 5. Stretching Increases Division Rate**

Dividing cells have large area, perimeter, and relative effective pressure.

(A) Division rate (percentage of cells entering mitosis per hour) increases in stretched tissue compared with unstretched. Ninety-five percent confidence intervals do not overlap, indicating significant difference. Each point represents the mean division rate from an animal cap.

(B) Percentage of cells that have undergone nuclear envelope breakdown (NEB) with respect to time in control stretched (red) and unstretched (blue) experiments from (A). Dashed lines indicate linear lines of best fit; control unstretched experiments have gradient 4.2% cells undergoing division per hour. Stretched experiments have initial gradient 8.1% and then 4.35% cells undergoing division per hour.

(C) Comparison of mean area of population of all cells versus dividing cells from unstretched and stretched control experiments. Error bars represent mean and 95% confidence intervals, which do not overlap between the population and dividing cells, indicating a significant difference.

(D) Comparison of mean perimeter of population of all cells versus dividing cells from unstretched and stretched control experiments. Error bars represent mean and 95% confidence intervals, which do not overlap between the population and dividing cells, indicating a significant difference.

(E) Heatmap showing predicted relative isotropic stress (effective pressure, $P^{eff}$) of dividing cells from control unstretched experiments. Areas and perimeters have been nondimensionalized using the preferred areas, $\tilde{A}_0$, fitted to each experiment in Figure S4C. Polygonal class (number of neighbors) indicated by marker color and style, with (4, 5, 6, 7, 8+) sided cells given in (blue, green, red, purple, yellow). Dashed vertical line represents mean area of all cells. Cells lying in red (blue) regions are under predicted net tension (compression).

divide, in contrast to division orientation, is directly sensitive to mechanical force.

## DISCUSSION

Previous models of cell division have demonstrated that specific features of cell shape, such as the cell cortex or TCJs, may be important in orienting the spindle (Bosveld et al., 2016; Hertwig, 1893; Luxenburg et al., 2011; Minc et al., 2011). We have presented a framework for characterizing cell shape in terms of its area, perimeter or TCJs (Methods S1). We find that the principal axis of shape defined by TCJs is the best predictor of division angle, better than cell shape as determined by area, perimeter, or a previous shape-sensing model based on microtubule length (Minc et al., 2011). Moreover, the principal axis of shape defined by TCJs aligns exactly with the principal axis of local stress (Nestor-Bergmann et al., 2018a), providing a non-invasive way to infer mechanical stress in individual cells in the epithelium. However, division angle is not better predicted in cells with higher or lower relative isotropic or shear stress, suggesting that cell-level mechanical stress is not a direct cue to orient the spindle. Our findings share similarities with observations in the *Drosophila* pupal notum, where TCJs have been hypothesized to localize force generators to orient the spindle (Bosveld et al., 2016). Notably, however, *Xenopus* animal cap cells do not undergo the dramatic mitotic rounding exhibited by cells in the notum.

Cell-cell adhesion has been linked to spindle orientation in MDCK cells, where E-cadherin instructs LGN/NuMA assembly at cell-cell contacts to orient divisions (Gloerich et al., 2017). E-cadherin polarizes along a stretch axis, reorienting divisions along this axis rather than according to cell shape (Hart et al., 2017). In accordance, we find division is less well predicted by shape in embryos injected with C-cadherin ΔC -6xmyc, lacking

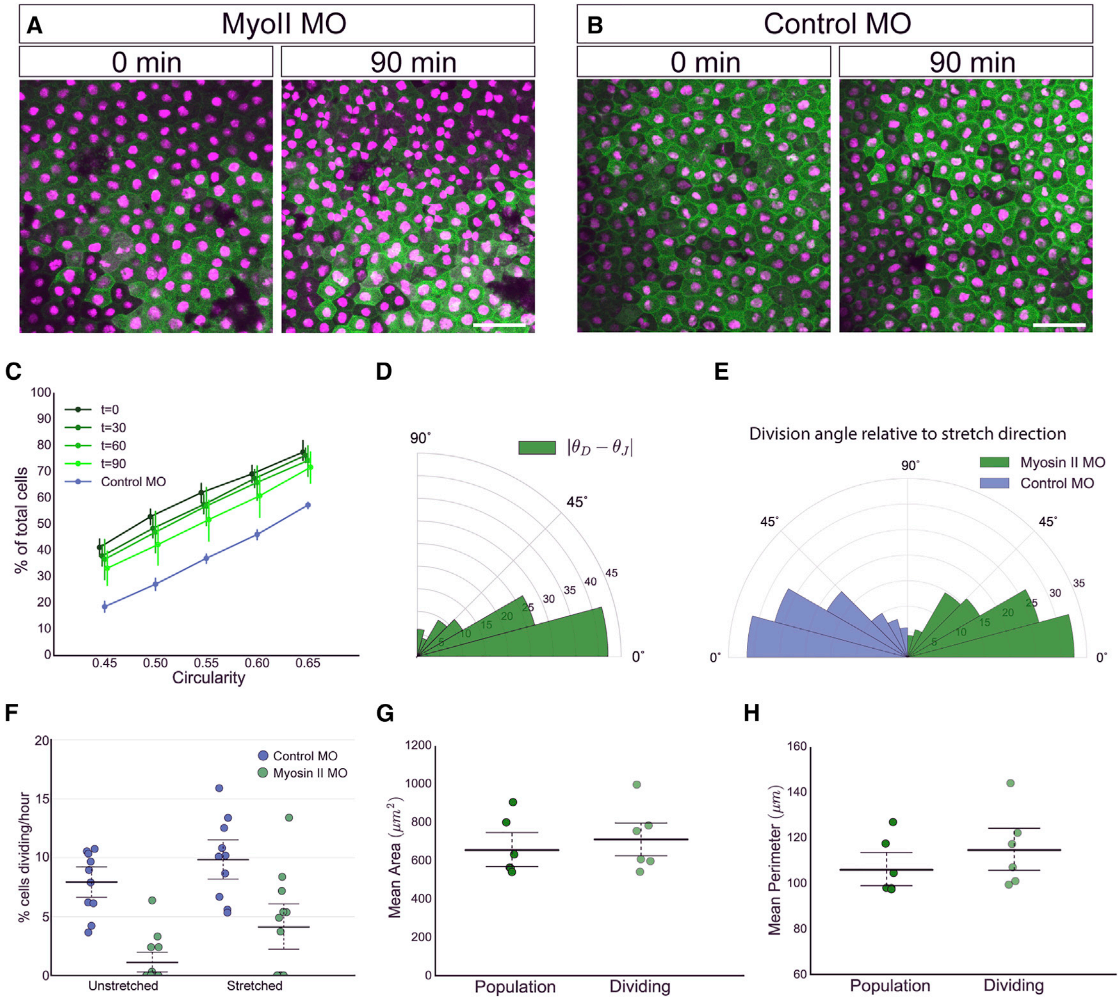


**Figure 6. Myosin II MO Cells Maintain Alignment of Division to TCJ Shape, but Have Perturbed Proliferation Rate**

(A) Images taken from a confocal time-lapse video of stretched myosin II morpholino-injected animal cap explants at 0 and 90 min intervals. Butterfly nuclei seen prominently at 90 min, where nuclei are in contact.

(B) Time-lapse images of control morpholino-injected stretched animal cap explants at 0 and 90 min intervals.

(C) Cumulative distribution of cell circularity defined by area, $C_A$, in myosin II MO knockdown stretched animal caps (shaded green) at t = 0, 30, 60, and 90 min after stretch. Cumulative distribution for unstretched t = 0 control MO knockdown experiments shown in blue. Error bars represent 95% confidence intervals. Error bars for myosin II MO t = 90 min distribution does not overlap with control MO, indicating a significant difference from unstretched shape. Markers are slightly offset for clarity.

(D) Rose plot of difference between division angle, $\theta_D$, and orientation of shape on the basis of junctions, $\theta_J$, for 216 cells from myosin II knockdown stretched experiments. Mann-Whitney U test found significant alignment compared with random ($p < 5.72 \times 10^{-15}$) but no significant difference from equivalent dataset in control stretched experiments. Percentages of cells shown.

(E) Rose plot of division angle relative to direction for stretch for control MO (532 cells; blue) and myosin II MO (301 cells; green) experiments. Mann-Whitney U and Kolmogorov-Smirnov tests found no significant difference between the two.

(F) Division rate (percentage of total cells entering mitosis per hour) in unstretched and stretched tissue from myosin II MO (green; n = 10 for unstretched and n = 12 for stretched) and control MO (blue; n = 13 for unstretched and n = 10 for stretched) experiments. Error bars represent mean and 95% confidence intervals.

(G) Comparison of mean area of population of all cells versus dividing cells from stretched myosin II knockdown experiments. Error bars represent mean and 95% confidence intervals, which overlap, indicating no significant difference.

(H) Comparison of mean perimeter of population of all cells versus dividing cells from stretched myosin II knockdown experiments. Error bars represent mean and 95% confidence intervals, which overlap, indicating no significant difference.

Scale bars in (A) and (B), 100 $\mu$m.

the cytosolic domain. Interestingly, overexpression of C-cadherin around the entire cell cortex leads to a switch in division orientation, from TCJs to division best predicted by a perimeter-based shape axis. As β-catenin is increased around the cell cortex when C-cadherin is overexpressed, we hypothesized that this may lead to altered recruitment of spindle orientation proteins, such as LGN and NuMA (Gloerich et al., 2017). Indeed, we find that although LGN is normally most highly localized to

TCJs, overexpression of C-cadherin leads to a loss of these "hotspots" and instead a more even spread of LGN around the entire cell perimeter. We suggest that in the wild-type situation, the hotspots of LGN localization at TCJs will recruit more NuMA and dynein providing localized force generation to orient the spindle according to TCJ shape, although this will need to be verified by further experimental work. Importantly, when C-cadherin is overexpressed, and the LGN hotspots are no longer present, we suggest that a perimeter-based shape sensing mechanism similar to that proposed by Minc et al. (2011) predominates. Furthermore, our results indicate that both shape-sensing mechanisms could be working in parallel in many cell shapes, where TCJ and perimeter shape are similar (as is most likely in more elongated cells), but that TCJ shape is most important in rounder cells (where perimeter and TCJ predictions of shape differ most greatly). Our TCJ-based system of spindle orientation is similar to the Mud-dependent TCJ-sensing mechanism in the *Drosophila* pupal notum (Bosveld et al., 2016). However, it is important to note a key difference: NuMA, the vertebrate homolog of *Drosophila* Mud, localizes to the nucleus during interphase, only localizing to the cortex after NEB (Bowman et al., 2006; Kiyomitsu and Cheeseman, 2012; Seldin et al., 2013; Gloerich et al., 2017). Of future interest will be to determine in vertebrate tissue how the TCJ localization of LGN influences the highly dynamic recruitment of NuMA. Indeed, recent work in MDCK cells has shown that on mitotic entry, when NuMA is released from the nucleus, it competes LGN away from E-cadherin at the cortex to locally form the LGN/NuMA complex (Gloerich et al., 2017); it will be important to determine if this is happening specifically at TCJs.

Stretching increases proliferation rate, which correlates with cell area, perimeter, and effective pressure. We see almost no proliferation in unstretched myosin II MO experiments, although, rather strikingly, the division rate is significantly increased following stretch. Dividing myosin II MO cells are not significantly larger in area or perimeter than the population as a whole, indicating that cell area has been decoupled as a division cue. Considering the established role of myosin II as a force generator (Clark et al., 2014; Gutzman et al., 2015; Vicente-Manzanares et al., 2009), it is possible that the myosin II MO cells cannot generate enough internal contractility in neighboring cells to engage the mechanical cues required for mitotic entry. Myosin II has also been shown to function in stress-sensing pathways (Hirata et al., 2015; Priya et al., 2015), which may explain why the proliferation rate in stretched myosin II MO cells does not reach the levels of stretched controls. Contrary to findings in other systems (Campinho et al., 2013), a global loss of myosin II does not alter division orientation relative to cell shape. However, future work should look to explore whether anisotropic biases in junctional myosin II affect division orientation, as was recently seen in the *Drosophila* germband (Scarpa et al., 2018).

In conclusion, we have combined whole-tissue stretching with a biomechanical model to propose separate roles for cell shape and mechanical stress in orienting the spindle and cueing mitosis. The mechanism involved in orienting the mitotic spindle does not appear to sense relative cell stress directly. Instead, division is best predicted by an axis of shape defined by TCJs and is dependent on functional cadherin and the recruitment of LGN. In contrast to this shape-based mechanism, we find that cells may directly sense mechanical stress as a cue for mitotic entry, in a myosin II-dependent manner.

## STAR★METHODS

Detailed methods are provided in the online version of this paper and include the following:

- KEY RESOURES TABLE
- CONTACT FOR REAGENT AND RESOURCE SHARING
- EXPERIMENTAL MODEL AND SUBJECT DETAILS
  - Xenopus laevis
- METHOD DETAILS
  - *Xenopus laevis* embryos and microinjection
  - Animal cap dissection and culture
  - Animal cap stretch manipulation and imaging
  - Immunofluorescence
  - Scanning EM
- QUANTIFICATION AND STATISTICAL ANALYSIS
  - Microtubule length-based division model
  - Implementation of the vertex-based model
  - Image analysis
  - Data analysis
- DATA AND SOFTWARE AVAILABILITY

## SUPPLEMENTAL INFORMATION

Supplemental Information can be found with this article online at https://doi.org/10.1016/j.celrep.2019.01.102.

## ACKNOWLEDGMENTS

A.N.-B. was supported by a Biotechnology and Biological Sciences Research Council (BBSRC) studentship. S.W., G.A.S.-V., and G.K.G. were supported by a Wellcome Trust / Royal Society Sir Henry Dale Fellowship to S.W. (098390/Z/12/Z), with additional funding from the Wellcome Trust Institutional Strategic Support Fund (ISSF) (105610/Z/14/Z). The Bioimaging Facility microscopes used in this study were purchased with grants from the BBSRC, the Wellcome Trust, and the University of Manchester Strategic Fund. We are grateful to Egor Zindy for his assistance in developing in-house nuclei tracking scripts. Thanks to Peter March and Roger Meadows for their help with the microscopy and to Lance Davidson for sharing cadherin constructs. Also, special thanks to Viki Allan, Tom Millard, and Nancy Papalopulu for their critical reading of the manuscript.

## AUTHOR CONTRIBUTIONS

Conceptualization, S.W. and O.E.J.; Methodology, A.N.-B., S.W., and O.E.J.; Software, A.N.-B.; Investigation, G.A.S.-V., G.K.G., T.S., A.N.-B., and S.W.; Formal Analysis, G.A.S.-V., G.K.G., and A.N.-B.; Writing – Original Draft, S.W., A.N.-B., G.A.S.-V., and G.K.G.; Writing – Review & Editing, G.A.S.-V., G.K.G., S.W., A.N.-B., and O.E.J.; Funding Acquisition, S.W. and O.E.J.; Supervision, S.W. and O.E.J.

## DECLARATION OF INTERESTS

The authors declare no competing interests.

Received: September 23, 2017
Revised: December 11, 2018
Accepted: January 28, 2019
Published: February 19, 2019

## REFERENCES

Benham-Pyle, B.W., Pruitt, B.L., and Nelson, W.J. (2015). Cell adhesion. Mechanical strain induces E-cadherin-dependent Yap1 and β-catenin activation to drive cell cycle entry. Science *348*, 1024–1027.

Bosveld, F., Markova, O., Guirao, B., Martin, C., Wang, Z., Pierre, A., Balakireva, M., Gaugue, I., Ainslie, A., Christophorou, N., et al. (2016). Epithelial tricellular junctions act as interphase cell shape sensors to orient mitosis. Nature *530*, 495–498.

Bowman, S.K., Neumüller, R.A., Novatchkova, M., Du, Q., and Knoblich, J.A. (2006). The Drosophila NuMA homolog Mud regulates spindle orientation in asymmetric cell division. Dev. Cell *10*, 731–742.

Brodland, G.W., Veldhuis, J.H., Kim, S., Perrone, M., Mashburn, D., and Hutson, M.S. (2014). CellFIT: a cellular force-inference toolkit using curvilinear cell boundaries. PLoS ONE *9*, e99116.

Campinho, P., Behrndt, M., Ranft, J., Risler, T., Minc, N., and Heisenberg, C.-P. (2013). Tension-oriented cell divisions limit anisotropic tissue tension in epithelial spreading during zebrafish epiboly. Nat. Cell Biol. *15*, 1405–1414.

Chiou, K.K., Hufnagel, L., and Shraiman, B.I. (2012). Mechanical stress inference for two dimensional cell arrays. PLoS Comput. Biol. *8*, e1002512.

Clark, A.G., Wartlick, O., Salbreux, G., and Paluch, E.K. (2014). Stresses at the cell surface during animal cell morphogenesis. Curr. Biol. *24*, R484–R494.

Davidson, L., von Dassow, M., and Zhou, J. (2009). Multi-scale mechanics from molecules to morphogenesis. Int. J. Biochem. Cell Biol. *41*, 2147–2162.

Effler, J.C., Kee, Y.-S., Berk, J.M., Tran, M.N., Iglesias, P.A., and Robinson, D.N. (2006). Mitosis-specific mechanosensing and contractile-protein redistribution control cell shape. Curr. Biol. *16*, 1962–1967.

Farhadifar, R., Röper, J.C., Aigouy, B., Eaton, S., and Jülicher, F. (2007). The influence of cell mechanics, cell-cell interactions, and proliferation on epithelial packing. Curr. Biol. *17*, 2095–2104.

Feroze, R., Shawky, J.H., von Dassow, M., and Davidson, L.A. (2015). Mechanics of blastopore closure during amphibian gastrulation. Dev. Biol. *398*, 57–67.

Finegan, T.M., Na, D., Cammarota, C., Skeeters, A.V., Nádasi, T.J., Dawney, N.S., Fletcher, A.G., Oakes, P.W., and Bergstralh, D.T. (2019). Tissue tension and not interphase cell shape determines cell division orientation in the *Drosophila* follicular epithelium. EMBO Journal *38*, e100072.

Fink, J., Carpi, N., Betz, T., Bétard, A., Chebah, M., Azioune, A., Bornens, M., Sykes, C., Fetler, L., Cuvelier, D., and Piel, M. (2011). External forces control mitotic spindle positioning. Nat. Cell Biol. *13*, 771–778.

Gloerich, M., Bianchini, J.M., Siemers, K.A., Cohen, D.J., and Nelson, W.J. (2017). Cell division orientation is coupled to cell-cell adhesion by the E-cadherin/LGN complex. Nat. Commun. *8*, 13996.

Gutzman, J.H., Sahu, S.U., and Kwas, C. (2015). Non-muscle myosin IIA and IIB differentially regulate cell shape changes during zebrafish brain morphogenesis. Dev. Biol. *397*, 103–115.

Hart, K.C., Tan, J., Siemers, K.A., Sim, J.Y., Pruitt, B.L., Nelson, W.J., and Gloerich, M. (2017). E-cadherin and LGN align epithelial cell divisions with tissue tension independently of cell shape. Proc. Natl. Acad. Sci. U S A *114*, E5845–E5853.

Heasman, J., Ginsberg, D., Geiger, B., Goldstone, K., Pratt, T., Yoshida-Noro, C., and Wylie, C. (1994). A functional test for maternally inherited cadherin in Xenopus shows its importance in cell adhesion at the blastula stage. Development *120*, 49–57.

Hertwig, O. (1893). Ueber den Werth der ersten Furchungszellen für die Organbildung des Embryo Experimentelle Studien am Frosch-und Tritonei. Archiv f. mikrosk. Anat. *42*, 662–807.

Hirata, H., Gupta, M., Vedula, S.R.K., Lim, C.T., Ladoux, B., and Sokabe, M. (2015). Actomyosin bundles serve as a tension sensor and a platform for ERK activation. EMBO Rep. *16*, 250–257.

Hoh, J.H., and Schoenenberger, C.A. (1994). Surface morphology and mechanical properties of MDCK monolayers by atomic force microscopy. J. Cell Sci. *107*, 1105–1114.

Hutson, M.S., Tokutake, Y., Chang, M.S., Bloor, J.W., Venakides, S., Kiehart, D.P., and Edwards, G.S. (2003). Forces for morphogenesis investigated with laser microsurgery and quantitative modeling. Science *300*, 145–149.

Ishihara, S., and Sugimura, K. (2012). Bayesian inference of force dynamics during morphogenesis. J. Theor. Biol. *313*, 201–211.

Jones, E., and Oliphant, E.P. (2001). SciPy: open source scientific tools for Python. http://www.scipy.org.

Jones, L.A., Villemant, C., Starborg, T., Salter, A., Goddard, G., Ruane, P., Woodman, P.G., Papalopulu, N., Woolner, S., and Allan, V.J. (2014). Dynein light intermediate chains maintain spindle bipolarity by functioning in centriole cohesion. J. Cell Biol. *207*, 499–516.

Joshi, S.D., and Davidson, L.A. (2010). Live-cell imaging and quantitative analysis of embryonic epithelial cells in Xenopus laevis. J. Vis. Exp. (*39*), 1949.

Kanda, T., Sullivan, K.F., and Wahl, G.M. (1998). Histone-GFP fusion protein enables sensitive analysis of chromosome dynamics in living mammalian cells. Curr. Biol. *8*, 377–385.

Keller, R.E. (1980). The cellular basis of epiboly: an SEM study of deep-cell rearrangement during gastrulation in Xenopus laevis. J. Embryol. Exp. Morphol. *60*, 201–234.

Keller, R.E., and Schoenwolf, G.C. (1977). An SEM study of cellular morphology, contact, and arrangement, as related to gastrulation inXenopus laevis. Wilhelm Roux Arch. Dev. Biol. *182*, 165–186.

Kiyomitsu, T., and Cheeseman, I.M. (2012). Chromosome- and spindle-pole-derived signals generate an intrinsic code for spindle position and orientation. Nat. Cell. Biol. *14*, 311–317.

Klymkowsky, M.W., and Hanken, J. (1991). Whole-mount staining of Xenopus and other vertebrates. Meth. Cell Biol. *36*, 419–441.

Kremer, J.R., Mastronarde, D.N., and McIntosh, J.R. (1996). Computer visualization of three-dimensional image data using IMOD. J. Struct. Biol. *116*, 71–76.

Kurth, T., Fesenko, I.V., Schneider, S., Münchberg, F.E., Joos, T.O., Spieker, T.P., and Hausen, P. (1999). Immunocytochemical studies of the interactions of cadherins and catenins in the early Xenopus embryo. Dev. Dyn. *215*, 155–169.

Landsberg, K.P., Farhadifar, R., Ranft, J., Umetsu, D., Widmann, T.J., Bittig, T., Said, A., Jülicher, F., and Dahmann, C. (2009). Increased cell bond tension governs cell sorting at the Drosophila anteroposterior compartment boundary. Curr. Biol. *19*, 1950–1955.

Lee, C.H., and Gumbiner, B.M. (1995). Disruption of gastrulation movements in Xenopus by a dominant-negative mutant for C-cadherin. Dev. Biol. *171*, 363–373.

Legoff, L., Rouault, H., and Lecuit, T. (2013). A global pattern of mechanical stress polarizes cell divisions and cell shape in the growing Drosophila wing disc. Development *140*, 4051–4059.

Luo, T., Mohan, K., Iglesias, P.A., and Robinson, D.N. (2013). Molecular mechanisms of cellular mechanosensing. Nat. Mater. *12*, 1064–1071.

Luxenburg, C., Pasolli, H.A., Williams, S.E., and Fuchs, E. (2011). Developmental roles for Srf, cortical cytoskeleton and cell shape in epidermal spindle orientation. Nat. Cell Biol. *13*, 203–214.

Mao, Y., Tournier, A.L., Bates, P.A., Gale, J.E., Tapon, N., and Thompson, B.J. (2011). Planar polarization of the atypical myosin Dachs orients cell divisions in Drosophila. Genes Dev. *25*, 131–136.

Miller, J.R., and Moon, R.T. (1997). Analysis of the signaling activities of localization mutants of beta-catenin during axis specification in Xenopus. J. Cell Biol. *139*, 229–243.

Minc, N., and Piel, M. (2012). Predicting division plane position and orientation. Trends Cell Biol. *22*, 193–200.

Minc, N., Burgess, D., and Chang, F. (2011). Influence of cell geometry on division-plane positioning. Cell *144*, 414–426.

Mishra, P., and Chan, D.C. (2014). Mitochondrial dynamics and inheritance during cell division, development and disease. Nat. Rev. Mol. Cell Biol. *15*, 634–646.

Nakagawa, S., and Cuthill, I.C. (2007). Effect size, confidence interval and statistical significance: a practical guide for biologists. Biol. Rev. Camb. Philos. Soc. *82*, 591–605.

Nestor-Bergmann, A., Goddard, G., and Woolner, S. (2014). Force and the spindle: mechanical cues in mitotic spindle orientation. Semin. Cell Dev. Biol. *34*, 133–139.

Nestor-Bergmann, A., Goddard, G., Woolner, S., and Jensen, O.E. (2018a). Relating cell shape and mechanical stress in a spatially disordered epithelium using a vertex-based model. Math. Med. Biol. *35* (*Suppl. 1*), 1–27.

Nestor-Bergmann, A., Johns, E., Woolner, S., and Jensen, O.E. (2018b). Mechanical characterization of disordered and anisotropic cellular monolayers. Phys. Rev. E *97*, 052409.

Pease, J.C., and Tirnauer, J.S. (2011). Mitotic spindle misorientation in cancer—out of alignment and into the fire. J. Cell Sci. *124*, 1007–1016.

Petridou, N.I., Stylianou, P., and Skourides, P.A. (2013). A dominant-negative provides new insights into FAK regulation and function in early embryonic morphogenesis. Development *140*, 4266–4276.

Priya, R., Gomez, G.A., Budnar, S., Verma, S., Cox, H.L., Hamilton, N.A., and Yap, A.S. (2015). Feedback regulation through myosin II confers robustness on RhoA signalling at E-cadherin junctions. Nat. Cell Biol. *17*, 1282–1293.

Quyn, A.J., Appleton, P.L., Carey, F.A., Steele, R.J.C., Barker, N., Clevers, H., Ridgway, R.A., Sansom, O.J., and Näthke, I.S. (2010). Spindle orientation bias in gut epithelial stem cell compartments is lost in precancerous tissue. Cell Stem Cell *6*, 175–181.

Scarpa, E., Finet, C., Blanchard, G., and Sanson, B. (2018). Actomyosin-driven tension at compartmental boundaries orients cell division independently of cell geometry in vivo. Dev. Cell *47*, 727–740.e6.

Schneider, C.A., Rasband, W.S., and Eliceiri, K.W. (2012). NIH Image to ImageJ: 25 years of image analysis. Nat. Methods *9*, 671–675.

Seldin, L., Poulson, N.D., Foote, H.P., and Lechler, T. (2013). NuMA localization, stability, and function in spindle orientation involve 4.1 and Cdk1 interactions. Mol. Biol. Cell *24*, 3651–3662.

Skoglund, P., Rolo, A., Chen, X., Gumbiner, B.M., and Keller, R. (2008). Convergence and extension at gastrulation require a myosin IIB-dependent cortical actin network. Development *135*, 2435–2444.

Sokac, A.M., Co, C., Taunton, J., and Bement, W. (2003). Cdc42-dependent actin polymerization during compensatory endocytosis in Xenopus eggs. Nat. Cell Biol. *5*, 727–732.

Starborg, T., Kalson, N.S., Lu, Y., Mironov, A., Cootes, T.F., Holmes, D.F., and Kadler, K.E. (2013). Using transmission electron microscopy and 3View to determine collagen fibril size and three-dimensional organization. Nat. Protoc. *8*, 1433–1448.

Stooke-Vaughan, G.A., Davidson, L.A., and Woolner, S. (2017). Xenopus as a model for studies in mechanical stress and cell division. Genesis *55*, e23004.

Streichan, S.J., Hoerner, C.R., Schneidt, T., Holzer, D., and Hufnagel, L. (2014). Spatial constraints control cell proliferation in tissues. Proc. Natl. Acad. Sci. U S A *111*, 5586–5591.

Sugimura, K., Lenne, P.-F., and Graner, F. (2016). Measuring forces and stresses in situ in living tissues. Development *143*, 186–196.

Théry, M., Pépin, A., Dressaire, E., Chen, Y., and Bornens, M. (2006). Cell distribution of stress fibres in response to the geometry of the adhesive environment. Cell Motil. Cytoskeleton *63*, 341–355.

Vicente-Manzanares, M., Ma, X., Adelstein, R.S., and Horwitz, A.R. (2009). Non-muscle myosin II takes centre stage in cell adhesion and migration. Nat. Rev. Mol. Cell Biol. *10*, 778–790.

Williams, M.E., Wilke, S.A., Daggett, A., Davis, E., Otto, S., Ravi, D., Ripley, B., Bushong, E.A., Ellisman, M.H., Klein, G., and Ghosh, A. (2011). Cadherin-9 regulates synapse-specific differentiation in the developing hippocampus. Neuron *71*, 640–655.

Woolner, S., and Papalopulu, N. (2012). Spindle position in symmetric cell divisions during epiboly is controlled by opposing and dynamic apicobasal forces. Dev. Cell *22*, 775–787.

Woolner, S., O'Brien, L.L., Wiese, C., and Bement, W.M. (2008). Myosin-10 and actin filaments are essential for mitotic spindle function. J. Cell Biol. *182*, 77–88.

Wyatt, T.P.J., Harris, A.R., Lam, M., Cheng, Q., Bellis, J., Dimitracopoulos, A., Kabla, A.J., Charras, G.T., and Baum, B. (2015). Emergence of homeostatic epithelial packing and stress dissipation through divisions oriented along the long cell axis. Proc. Natl. Acad. Sci. U S A *112*, 5726–5731.

Xu, G.-K., Liu, Y., and Li, B. (2015). How do changes at the cell level affect the mechanical properties of epithelial monolayers? Soft Matter *11*, 8782–8788.

## STAR★METHODS

### KEY RESOURES TABLE

| REAGENT or RESOURCE | SOURCE | IDENTIFIER |
|---|---|---|
| Antibodies | | |
| Rabbit anti-β-catenin | Abcam | Catalogue number: ab2365; RRID: AB_303014 |
| Mouse anti c-myc 9E10 | Santa-cruz | Catalogue number: sc-40; RRID: AB_627268 |
| Alexa Fluor 488 Goat Anti-Rabbit IgG (H+L) Antibody | Life technologies | Catalogue number: A11008; RRID: AB_143165 |
| Alexa Fluor 568 Goat Anti-Mouse IgG (H+L) Antibody | Life technologies | Catalogue number: A11004; RRID: AB_2534072 |
| Bacterial and Virus Strains | | |
| Subcloning Efficiency DH5α Competent Cells | Thermo Fisher Scientific | 18265017 |
| Chemicals, Peptides, and Recombinant Proteins | | |
| Silicone Sylgard 184 Kit 1.1Kg | Scientific Laboratory Supplies | 63416.5S |
| PMSG-Intervet (Pregnant Mare Serum Gonadotrophin) | Intervet UK | N/A |
| Chorulon (Human Chorionic Gonadotrophin) | Intervet UK | N/A |
| MS222 – Ethyl 3-aminobenzoate methanesulfonate salt | Merck | A5040-100G |
| Phenol:Chloroform:IAA, 25:24:1 | Thermo Fisher Scientific | AM9730 |
| Fibronectin bovine plasma | Merck | F1141-1MG |
| NotI | New England Biolabs | R0189L |
| Critical Commercial Assays | | |
| mMessage mMachine SP6 transcription kit | Life Technologies | AM1340 |
| PureLink Quick Plasmid Miniprep Kit | Life Technologies | K210010 |
| Experimental Models: Organisms/Strains | | |
| Mature female *Xenopus laevis* Albino and Pigmented | Bred in-house and from European *Xenopus* Resource Centre (EXRC). | https://xenopusresource.org/ |
| Mature male *Xenopus laevis* Albino and Pigmented | Bred in-house and from European *Xenopus* Resource Centre (EXRC). | https://xenopusresource.org/ |
| Oligonucleotides | | |
| Morpholino: MHC-B (Myosin Heavy Chain-B, myosin II) 5′-CTTCCTGCCCTGGTCTCTGTGACAT-3′ | Skoglund et al., 2008. (Gene Tools LLC) | N/A |
| Morpholino: Vinculin MO 5′-TATGGAAGACCGGCATC TTGGCAAT-3′ | Petridou et al., 2013 (Gene Tools LLC) | N/A |
| Morpholino: Standard control 5′-CCTCTTACCTCAGTT ACAATTTATA-3′ | Gene Tools LLC | Product name "Standard Control oligo" |
| Recombinant DNA | | |
| mCherry-Histone2B in pCS2+ | Kanda et al., 1998 (GFP-Histone2B) | N/A |
| GFP-α-tubulin in pCS2+ | Woolner et al., 2008 | |
| Cadherin 3a full length:6x myc-tag in pCS2+ | Kurth et al., 1999 (A gift from Lance Davidson) | N/A |
| Cadherin 3a deleted cytosolic domain: 6x myc-tag in pCS2+ | Kurth et al., 1999 (A gift from Lance Davidson) | N/A |
| β-catenin-GFP in pCS2+ | Randall Moon (Miller and Moon, 1997). | Addgene plasmid #16839 |
| GFP-LGN in pBABE (subcloned into pCS2+ vector). | Iain Cheeseman (Kiyomitsu and Cheeseman, 2012). | Addgene plasmid #37360 |
| Software and Algorithms | | |
| ImageJ 1.51a (straight line tool, ROI manager) | NIH Schneider et al., 2012 | https://imagej.nih.gov/ij/ |
| Imaris version 7.6.5 | Bitplane | http://www.bitplane.com/imaris |

(*Continued on next page*)

*Continued*

| REAGENT or RESOURCE | SOURCE | IDENTIFIER |
|---|---|---|
| GraphPad Prism 7 | GraphPad Software | https://www.graphpad.com/scientific-software/prism/ |
| Python v3.6.5 – in-house python scripts implementing watershed algorithm. | Python Core Team | https://www.python.org/ |
| Vertex-based model | Nestor-Bergmann et al., 2018a (section 3.8) | N/A |
| SciPy library (for statistical tests) | Jones and Oliphant, 2001 | N/A |

## CONTACT FOR REAGENT AND RESOURCE SHARING

Further information and requests for resources and reagents should be directed to and will be fulfilled by the Lead Contact, Sarah Woolner (sarah.woolner@manchester.ac.uk).

## EXPERIMENTAL MODEL AND SUBJECT DETAILS

### Xenopus laevis

Female pigmented and albino *Xenopus laevis* were housed within tanks maintained by the in-house animal facility at the University of Manchester. These females were used for embryo collection only. Frogs were pre-primed 4-7 days in advance of egg collection with 50 U of pregnant mare serum gonadotrophin (Intervet UK) and then primed with 500 U of human chorionic gonadotrophin (Intervet UK) 18 hours before use. Primed frogs were maintained in individual tanks containing Marc's modified Ringer's (MMR; 100mM NaCl, 2mM KCl, 1mM MgCl2, and 5mM HEPES, pH7.4). *In vitro* fertilization was performed by swirling mashed testis through the eggs within a Petri dish. Male frogs were only used for testis extraction (in which males were euthanized by injection of MS222 (Tricaine) into the dorsal lymph sac to induce terminal anesthesia). All *Xenopus* work was performed using protocols approved by the UK Government Home Office and covered by Home Office Project License PFDA14F2D (License Holder: Professor Enrique Amaya) and Home Office Personal Licenses held by Sarah Woolner, Georgina Stooke-Vaughan and Georgina Goddard.

## METHOD DETAILS

### *Xenopus laevis* embryos and microinjection

*Xenopus laevis* embryos were obtained and injected as described previously (Woolner and Papalopulu, 2012). RNA was synthesized as described previously (Sokac et al., 2003) and microinjected at the following needle concentrations: 0.5 mg/ml GFP-$\alpha$-tubulin; 0.1 mg/ml cherry-histone2B(Kanda et al., 1998); 0.125 mg/ml cadherin 3a full length:6x myc-tag; 0.125 mg/ml cadherin 3a deleted cytosolic domain:6x myc-tag (Kurth et al., 1999). For mosaic expression of $\beta$-catenin-GFP (Addgene plasmid #16839, Randall Moon) and GFP-LGN (sub-cloned into pCS2+ from Addgene plasmid #37360, Iain Cheeseman), RNA was injected into a single cell at the 4-cell stage at 0.25 mg/ml (needle concentration). Morpholinos prepared as 1mM stocks (diluted in water) were heated at 65°C for 5 minutes and microinjected at a needle concentration of 1mM and needle volume of 2.5nl into all cells of four-cell stage embryos. The MOs used were MHC-B (Myosin Heavy Chain-B, myosin II) MO (5′-CTTCCTGCCCTGGTCTCTGTGACAT-3′; (Skoglund et al., 2008) and standard control MO (5′-CCTCTTACCTCAGTTACAATTTATA-3′; Gene Tools LLC). All embryos were incubated at 16°C for approximately 20 hours prior to animal cap dissection.

### Animal cap dissection and culture

Animal cap tissue was dissected from the embryo at stage 10 of development (early gastrula stage) following a previously described protocol (Joshi and Davidson, 2010), and cultured in Danilchik's for Amy explant culture media (DFA; 53mM $NaCl_2$, 5mM $Na_2CO_3$, 4.5mM Potassium gluconate, 32mM Sodium gluconate, 1mM $CaCl_2$, 1mM $MgSO_4$) on a 20mm × 20mm elastomeric PDMS (Sylgard 184, SLS) membrane made in a custom mold and coated with fibronectin (fibronectin from bovine plasma, Merck). Explants were held in place by a coverslip fragment. Each membrane was then incubated at 18°C for at least 2 hours prior to imaging.

### Animal cap stretch manipulation and imaging

Each PDMS membrane was attached to a stretch apparatus (custom made by Deben UK Limited) fixed securely to the stage of a Leica TCS SP5 AOBS upright confocal and a 0.5mm (to remove sag on the membrane) or 8.6mm uniaxial stretch was applied for unstretched and stretched samples respectively. Images were collected on a Leica TCS SP5 AOBS upright confocal using a 20x/0.50 HCX Apo U-V-I (W (Dipping Lens)) objective and 2x confocal zoom. The distance between optical sections was maintained at 5 $\mu$m and the time interval between each frame was 20 s, with each sample being imaged for up to 2.5 hours. For quantification of $\beta$-catenin-GFP and GFP-LGN localization, animal caps were prepared as described but timelapse movies were collected with 2$\mu$m

optical sections and a time interval of 1 minute between frames. Maximum intensity projections of these 3D stacks are shown in the results; except for the GFP-LGN timelapse (Figure 4G), which is an average intensity projection.

### Immunofluorescence

Embryos were fixed at stage 12 following the protocol previously detailed by Jones et al., (2014) (Jones et al., 2014). Embryos were incubated in primary and secondary antibodies in TBSN/BSA (Tris- buffered saline: 155mM NaCl, 10mM Tris-Cl [pH 7.4]; 0.1% Nonidet P-40; 10 mg/ml BSA) overnight at 4°C, with five 1 hour washes with TBSN/BSA following each incubation. Primary antibodies were: anti-β-catenin at 1:200 dilution, raised in rabbit (Abcam) and anti *c-myc* 9E10 at 1:1000 dilution, raised in mouse (Santa-cruz). Alexa Fluor secondary antibodies, anti-rabbit 488 and anti-mouse 568 (Life Technologies) were used at a dilution of 1:400. After staining, embryos were methanol dehydrated, then cleared and mounted in Murray's Clear (2:1, benzyl benzoate:benzyl alcohol; (Klymkowsky and Hanken, 1991)). Images were collected on a Leica TCS SP5 AOBS inverted confocal using a 63x HCX PL APO (Oil λBL) objective and 1024 × 1024 format. Single confocal slices are shown in the results.

### Scanning EM

Uninjected embryos were allowed to develop to stage 10 at 16°C and then animal cap tissue was dissected and allowed to adhere to a fibronectin PDMS membrane as described previously. After 2 hours the animal caps were fixed following a protocol previously detailed by Jones et al., 2014. Briefly, the animal caps were fixed in 3.7% PFA and 2.5% Glutaraldehyde in BRB80 buffer (80mM PIPES, 1mM $MgCl_2$, 1mM EGTA, pH 6.8) overnight at 4°C. Samples were processed using a high density staining method detailed in full by Williams et al., 2011 (supplementary protocol), but briefly comprising a 1 hour fix in 2% (wt/vol) osmium tetroxide and 1.5% (wt/vol) potassium ferrocyanide in 0.1M cacodylate buffer. This was followed by a 20 minute incubation in 1% (wt/vol) thiocarbohydrazide and a 30 minute incubation in 2% (wt/vol) osmium teroxide, followed by a final incubation in 1% (wt/vol) uranyl acetate overnight at 4°C. Samples were then stained with freshly prepared Walton's lead aspartate (0.02M lead nitrate and 0.03M in aspartic acid, adjusted to pH 5.5) for 30 minutes at 60°C prior to dehydration, embedding in Epon 812 (hard formulation), and trimming on a standard microtome. Samples were visualized using a microtome (3View; Gatan) within a Quanta 250 FEG; FEI scanning electron microscope using the following imaging conditions: indicated quadrant magnification of 1600x, accelerating voltage of 3.8kV, pressure at 0.33 Torr. Images were collected at 4000 × 5000 pixels with a dwell time of 10 μs. Raw data was converted to an MRC file stack using IMOD (Kremer et al., 1996; Starborg et al., 2013) and further processed using Imaris software (Bitplane).

## QUANTIFICATION AND STATISTICAL ANALYSIS

### Microtubule length-based division model

Details of the model and implementation are given in Methods S1 of the Supplemental Information. The predicted torques and corresponding division axes were calculated using in-house Python scripts that are available upon request.

### Implementation of the vertex-based model

The numerical simulations of the vertex-based model were carried out using the same scripts outlined in section 3.8 of Nestor-Bergmann et al. (2018a). Model parameters used for all simulations were $(\Lambda, \Gamma) = (-0.259, 0.172)$, determined using a fitting procedure described in Nestor-Bergmann et al. (2018a).

### Image analysis

Image analysis was performed using ImageJ (Schneider et al., 2012). Cell division orientation was quantified using the straight-line tool to draw a line between the dividing nuclei of a cell in late anaphase (a stage in mitosis where division orientation is set and the spindle undergoes no further rotation (Woolner et al., 2008; Woolner and Papalopulu, 2012)). Using the ROI manager the angle of division relative to stretch (horizontal axis) was recorded along with the frame and location of the division. Single cell edges and junctions were manually traced 40 s before NEB using the freehand paintbrush tool. The whole population of cells in the apical layer of the animal cap was manually traced, along with peripheral junctions and cell centers, using the freehand paintbrush tool. Segmentation of the cell boundaries was performed using in-house Python scripts implementing a watershed algorithm. Geometric features of the cells, such as area and perimeter, were extracted and analyzed in Python; for further details on how cell shape was characterized using the segmented images, please see Supplemental Information, Methods S1. To quantify β-catenin-GFP and GFP-LGN localization at TCJs in mitotic cells, movies of unstretched and stretched animal caps were analyzed as follows: mitotic cells which had non-expressing neighbors were selected at early metaphase. A single optical slice which was level with the center of metaphase nuclei (visualized by mCherry-H2B) was selected and ROI's were drawn around TCJs and the corresponding cell edges in ImageJ. Mean gray values were measured for each ROI and TCJ and cell edge gray values were averaged (mean) for each cell. A ratio between average TCJ intensity and average cell edge intensity (Mean TCJ intensity/Mean cell edge intensity) for each mitotic cell was then calculated.

### Data analysis

The data analysis and plotting was carried out using in-house Python scripts. Statistical tests were performed using the SciPy library (Jones and Oliphant, 2001) and Prism 7 (GraphPad Software, Inc). Mann-Whitney U tests were used to assess if rose histograms were distributed closer to zero. Kolmogorov-Smirnov tests were used to assess if two distributions were significantly different. Otherwise, bootstrapping with 95% confidence intervals, which allow the precision of the estimate to be seen (Nakagawa and Cuthill, 2007), were used to assess significance. All statistical analysis is shown within the main text and corresponding figure legends.

## DATA AND SOFTWARE AVAILABILITY

Implementation of the microtubule division model can be downloaded from https://github.com/Alexander-Nestor-Bergmann/Minc_division_model. All other data processing scripts and implementation of the vertex-based model are available upon request.

Cell Reports, Volume *26*

## Supplemental Information

# Decoupling the Roles of Cell Shape and Mechanical Stress in Orienting and Cueing Epithelial Mitosis

**Alexander Nestor-Bergmann, Georgina A. Stooke-Vaughan, Georgina K. Goddard, Tobias Starborg, Oliver E. Jensen, and Sarah Woolner**

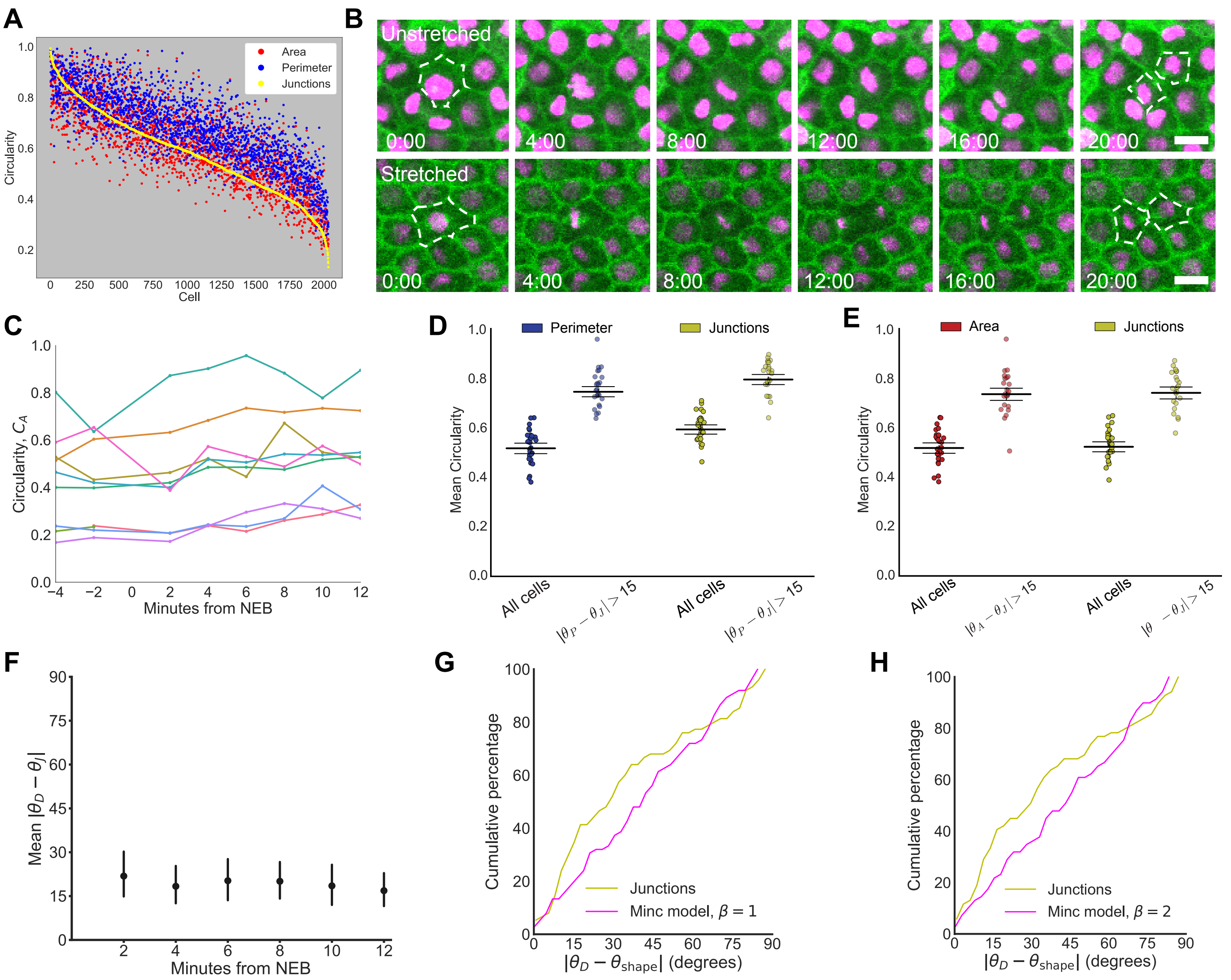


**Figure S1. Related to Figure 2. A.** Circularities of 2035 cells from unstretched experiments, with shape characterised by area, perimeter and junctions plotted in red, blue and yellow respectively. Cells have been ordered in descending order of junction-based circularity ($C_J$), with the corresponding values of $C_A$ and $C_P$ plotted alongside. **B.** Images taken from a confocal timelapse movie of a division in unstretched (upper) and stretched (lower) tissue from NEB to formation of cleavage plane. Cells do not show any significant rounding during this time period. **C.** Line plots showing circularity defined by cell area, $C_A$, against time (in minutes) since NEB for 9 example cells from stretched tissue. Lines show no significant trend of increasing or decreasing circularity. **D.** Mean circularity of subpopulation used in Figure 3E (darker points) vs mean circularity of all cells (lighter points), where shape is characterised by perimeter (blue) and junctions (yellow). Error bars represent mean and 95% confidence intervals. **E.** Equivalent plot to D, but shape characterised by area in red and junctions in yellow. **F.** Alignment of division orientation with junctional shape at different timepoints (minutes from NEB; n = 162 cells). **G.** Cumulative plot of difference between division angle, $\theta_D$, and orientation of shape based on the Minc model when $\beta = 1$ (magenta; $\theta_{shape} = \theta_{Minc}$; Minc et al., 2011) and junctions (yellow; $\theta_{shape} = \theta_J$). **H.** As G but $\beta = 2$.

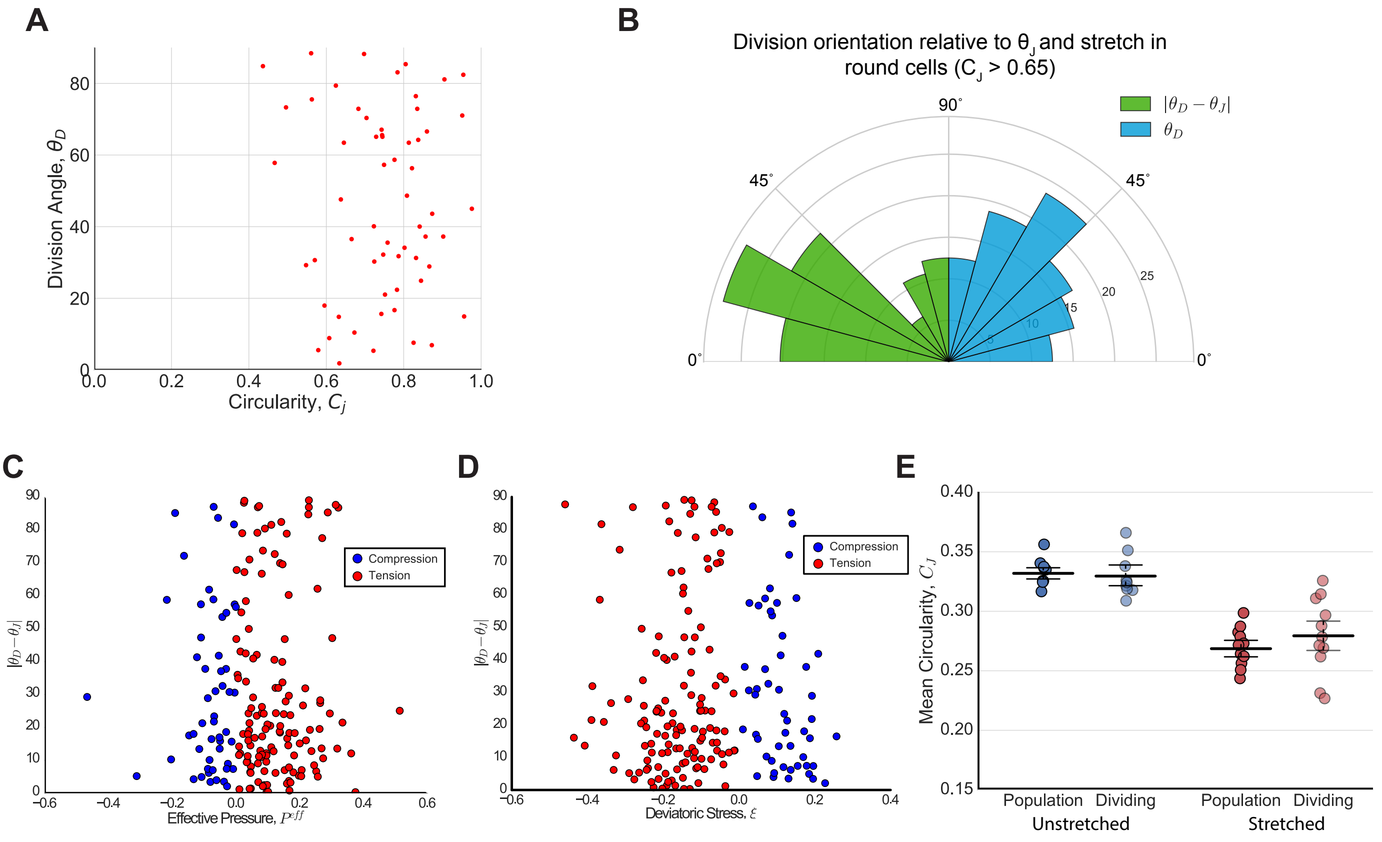


**Figure S2. Related to Figure 3: A.** Scatter plot of division angle relative to stretch direction ($\theta_D$) vs cell circularity, $C_J$, showing no significant relationship. **B.** Rose histogram showing division orientation relative to TCJ shape ($|\theta_D - \theta_J|$) and the stretch axis ($\theta_D$) in round cells ($C_J$ > 0.65). **C.** Correlation of effective pressure vs $|\theta_D - \theta_J|$. Spearman rank correlation coefficient found no significant correlation. Cells under tension (compression) plotted in red (blue). **D.** Correlation of shear stress vs $|\theta_D - \theta_J|$. Spearman rank correlation coefficient found no significant correlation. Cells under tension (compression) plotted in red (blue). **E.** Comparison of mean circularity, $C_J$, of population of all cells vs dividing cells from unstretched and stretched control experiments. Error bars represent mean and 95% confidence intervals, which overlap between the population and dividing cells, indicating no significant difference.

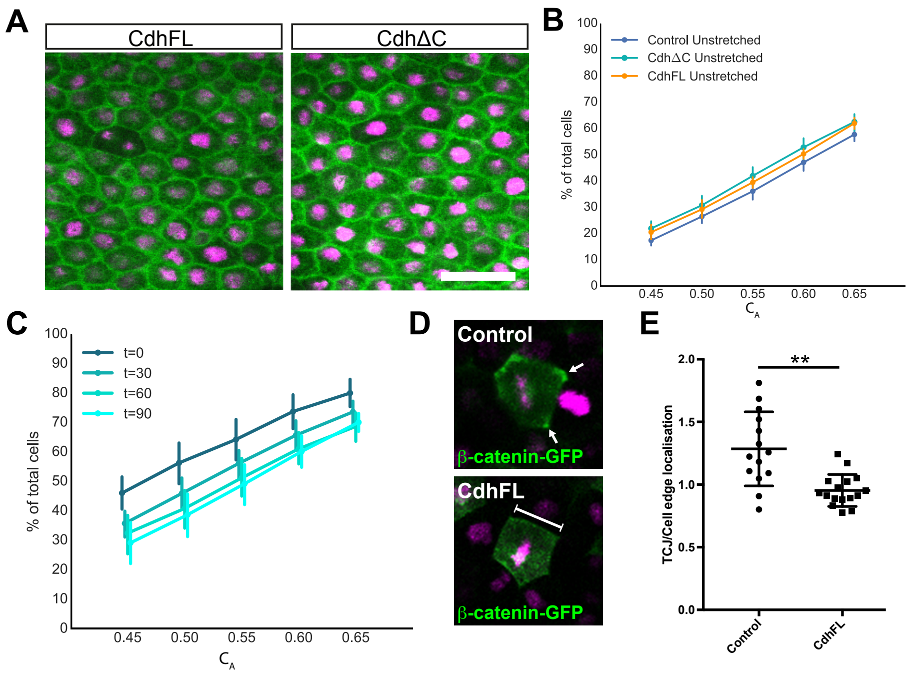


**Figure S3. Related to Figure 4: A.** Images taken from a confocal timelapse movie of CdhFL- (left) and CdhΔC- (right) injected stretched animal cap explants (green: GFP-alpha-tubulin; magenta: cherry-histone2B). Scale bar 50μm. **B.** Cumulative plots of cell circularity defined by area, $C_A$, in unstretched CdhΔC-injected (cyan), CdhFL-injected (orange) and control (blue) animal caps. Error bars represent 95% confidence intervals, which overlap indicating no significant difference. **C.** Cumulative plots of cell circularity defined by area, $C_A$, in CdhΔC-injected stretched animal caps at 0, 30, 60 and 90 mins after stretch (stretch applied just before 0 min). 100% of cells have $C_A < 1$. **D.** Image taken from time-lapse movies of control and CdhFL-injected animal cap tissue expressing β-catenin-GFP in a mosaic fashion. In control cells, β-catenin-GFP is enriched at TCJs during mitosis (arrows). The enrichment of β-catenin-GFP at TCJs is lost when CdhFL is expressed, with localisation spread throughout the cell edge (line). F. Quantification of β-catenin-GFP localization at TCJs compared to cell edges in single mitotic cells in animal caps. β-catenin-GFP is more strongly localised at TCJs compared to cell edges in controls but this bias is lost in CdhFL-injected tissue (*$p < 0.01$ Komogorov-Smirnov test; n = 14, 16 mitotic cells from 6, 4 unrelated animal caps for control and CdhFL, respectively).

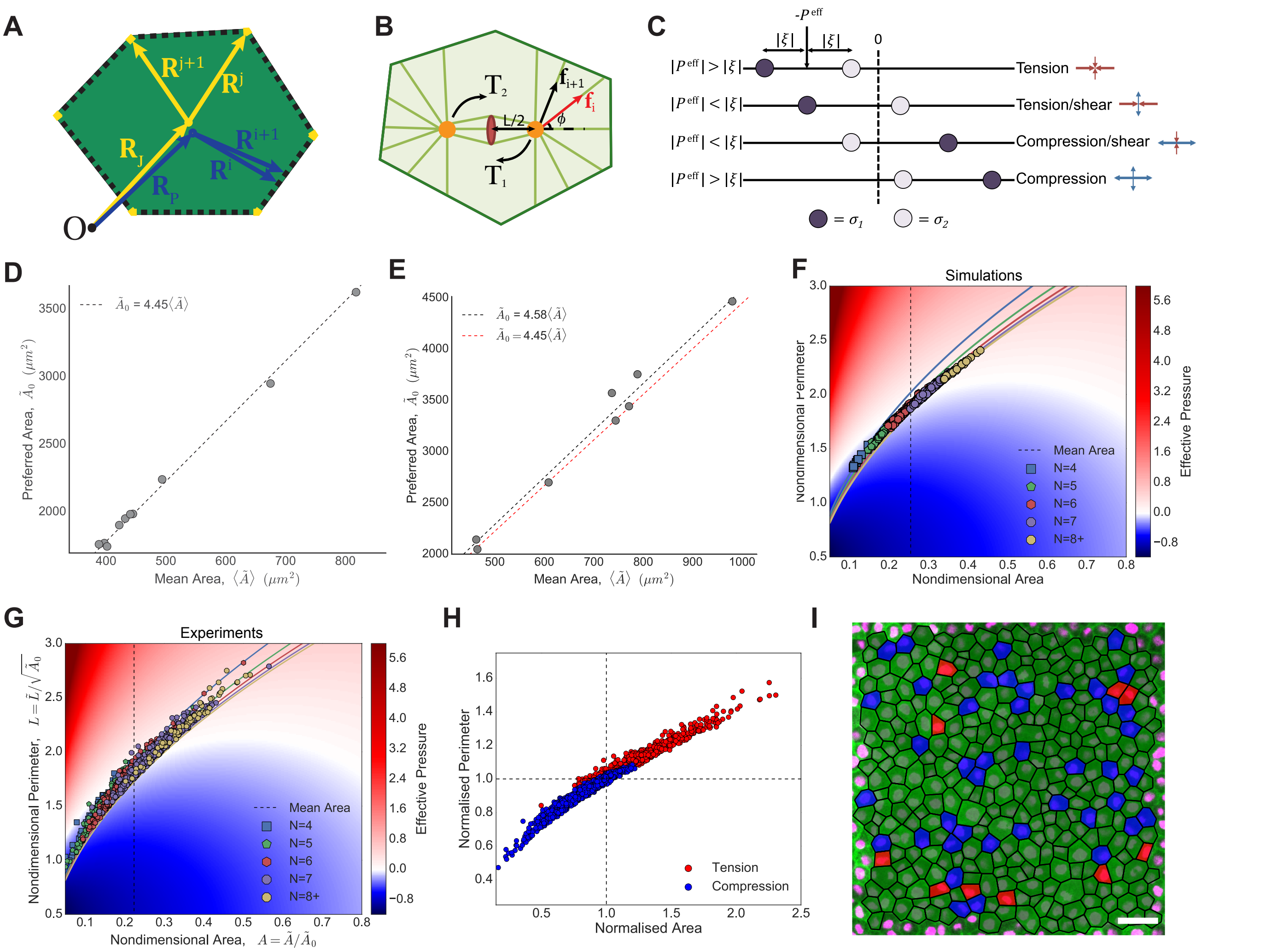


**Figure S4. Related to STAR Methods: A.** Representation of cell geometry outlined in STAR methods and Methods S1. Cells are computationally segmented such that their boundary pixels (black dots) and tricellular junctions (yellow dots) are extracted. Vectors based on cell perimeter (tricellular junctions) given in blue (yellow), with the centroid labeled $R_P$, ($R_J$). **B.** Shape-based division model proposed by Minc et al., 2011. Microtubules (light green lines) exert length-dependent forces on the spindle about the centrosomes (orange dots), exerting a net torque and rotating the spindle. **C**.Four possible cases of cell stress, based on the eigenvalues of the stress tensor. Horizontal lines represent a shared real number line with dark (light) circles indicating the value of $\sigma_1$ ($\sigma_2$). Vertical dashed line marks the location of zero. Arrows on far right represent direction of principal axes of stress, with red (blue) lines indicating tension (compression). **D**. Mean cell areas, $\langle \tilde{A} \rangle$, from 10 animal cap explants plotted against the preferred area, $\tilde{A}_0$, fitted to each experiment. Cells were polygonised (straight edges drawn between tricellular junctions) before the areas were computed, in order to be equivalent to the vertex-based model. Dashed line indicates linear line of best fit. **E.** Mean cell areas, $\langle \tilde{A} \rangle$, plotted against the preferred area, $\tilde{A}$, from 8 myosin II MO animal cap explants. Black (red) dashed line indicates linear line of best fit for myosin II MO (control, given in D) experiments. ANCOVA test suggests that slope of myosin II MO cells is not significantly different to control. **F.** Heat map showing relative effective pressure, $P^{eff}$, of a realisation of 800 simulated cells in a periodic box, with parameters ($\Lambda$,$\Gamma$) = (-0.259, 0.172), under conditions of zero global stress. Cell polygonal class indicated by marker colour and style, with (4,5,6,7,8+) sided cells given in (blue, green, red, purple, yellow). Equivalently coloured lines indicate exact perimeter-area relationship for perfect polygons. Dashed vertical line represents mean area of all cells. Cells lying in red (blue) regions are under net tension (compression). **G.** Equivalent plot to F for experimental control unstretched population data. Cell areas and perimeters have been nondimensionalised using the preferred area, $\tilde{A}_0$, such that the relative effective pressure may be calculated. A separate $\tilde{A}_0$ was fitted to each experiment, to account for differences in average cell size across experiments (data shown in Figure S4D). **H.** Normalised area vs normalised perimeter for the experimental cells given in G. Cell area and perimeter were normalised relative to the mean of each experiment. Cells under net tension (compression) are plotted in red (blue). Dashed vertical (horizontal) line represents cell area (perimeter) equal to the mean. **I.** Example segmented cells from an unstretched experiment, demonstrating where area is a poor predictor of effective pressure. Cells in red (blue) are predicted to be under tension (compression), but have area smaller (larger) than the mean. Scale bar 50µm.

# Methods S1. Related to STAR Methods

## 1 Deriving cell shape tensors

We characterise cell shape by deriving three shape tensors based on the distribution of cell area, perimeter and tricellular junctions. The shape tensors are defined via the cells' second moments of area, perimeter or tricellular junctions and so each is associated with a different centre of mass, which is defined in terms of the first moments of area, perimeter or tricellular junctions respectively. We provide equations for calculating the centroid using each measure; the centroid may also be calculated as the arithmetic mean position of all the points used to define each measure.

Cells from the confocal imaging data are computationally segmented, giving $N_C$ cells labelled with greek subscripts $\alpha = 1, 2, \ldots, N_C$. Each cell is defined by $P$ pixels, labelled anticlockwise $i = 0, 1, \ldots, P-1$, representing the cell cortex, with $Z$ tricellular junctions (pixels shared by three cells; we include the rare cases in which pixels are shared by more than three cells), labelled anticlockwise $j = 0, 1, \ldots, Z-1$. Boundary pixels are labelled with Latin super/sub-scripts, $i$, and tricellular junctions with Latin super/sub-scripts, $j$. Subscripts denote vectors originating from the origin (i.e. $\mathbf{R}_i$) and superscripts denote vectors originating from cell centroids (i.e. $\mathbf{R}^i$). Tangents between pixels are denoted $\mathbf{t}^i = \mathbf{R}^{i+1} - \mathbf{R}^i$. An image detailing the geometry of the system is given in Supplemental Figure 4A.

### 1.1 Tricellular junction-based centroid and shape tensor

The tricellular junction-based centroid, $\mathbf{R}_J$, is the arithmetic mean position of the cell's tricellular junctions. For a cell with $Z$ tricellular junctions labelled $j = 0, \ldots, Z-1$ we have

$$\mathbf{R}_J = \frac{1}{Z} \sum_{j=0}^{Z-1} \mathbf{R}_j \tag{1.1}$$

where $\mathbf{R}_j$ is the vector running from the origin to tricellular junction $j$.

Let $\mathbf{R}^j = \mathbf{R}_j - \mathbf{R}_J$ be the vector running from the cell centroid to tricellular junction $j$. We define the tricellular junction-based shape tensor as

$$\mathsf{S}_J = \frac{1}{Z} \sum_{i=0}^{Z-1} \mathbf{R}^i \mathbf{R}^i, \tag{1.2}$$

where $\mathbf{R}^i \mathbf{R}^i$ is a vector outer product.

### 1.2 Perimeter-based centroid and shape tensor

The perimeter-based centroid, $\mathbf{R}_P$, of cell $\alpha$, is classically defined as

$$\mathbf{R}_P = \frac{\int_{\partial\alpha} \mathbf{R}\, \mathrm{d}s}{\int_{\partial\alpha} \mathrm{d}s}, \tag{1.3}$$

where the numerator is the first moment of perimeter, $\mathbf{R}$ represents vectors across the continuous cell perimeter from the origin, $\mathrm{d}s$ is a line element and the integral is around the cell perimeter, $\partial\alpha$. For a polygonised segmented cell, we can write

$$\mathbf{R} = \mathbf{R}^i + b\mathbf{t}^i \tag{1.4}$$

and

$$ds = |\mathbf{t}^i|\, db = l^i\, db, \tag{1.5}$$

where $0 \le b \le 1$ and $l^i$ is the length of the straight line connecting pixels $i$ and $i+1$. Thus (1.3) becomes

$$\begin{aligned}\mathbf{R}_P &= \frac{\sum\limits_{i=0}^{Z-1} \int_0^1 \left[\mathbf{R}^i + b(\mathbf{R}^{i+1} - \mathbf{R}^i)\right] l^i\, db}{\sum\limits_{i=0}^{Z-1} l^i\, db} \\ &= \frac{1}{L} \sum_{i=0}^{Z-1} \frac{l^i}{2} \left(\mathbf{R}^i + \mathbf{R}^{i+1}\right),\end{aligned} \tag{1.6}$$

where $L = \sum\limits_{i=0}^{Z-1} l^i$ is the perimeter of the cell.

We define the perimeter-based shape tensor, $\mathsf{S}_P$, via the second moment of perimeter as

$$\mathsf{S}_P = \int_{\partial\alpha} (\mathbf{R} - \mathbf{R}_P)(\mathbf{R} - \mathbf{R}_P)\, ds. \tag{1.7}$$

Using (1.4) and (1.5), this becomes

$$\begin{aligned}\mathsf{S}_P &= \sum_{i=0}^{P-1} \int_0^1 \left[\mathbf{R}^i\mathbf{R}^i + b(\mathbf{t}^i\mathbf{R}^i + \mathbf{R}^i\mathbf{t}^i) + b^2\mathbf{t}^i\mathbf{t}^i\right] l^i\, db \\ &= \sum_{i=0}^{P-1} \left[\mathbf{R}^i\mathbf{R}^i + \tfrac{1}{2}(\mathbf{t}^i\mathbf{R}^i + \mathbf{R}^i\mathbf{t}^i) + \tfrac{1}{3}\mathbf{t}^i\mathbf{t}^i\right] l^i \\ &= \sum_{i=0}^{P-1} l^i \left[\tfrac{1}{3}(\mathbf{R}^{i+1}\mathbf{R}^{i+1} + \mathbf{R}^i\mathbf{R}^i) + \tfrac{1}{6}(\mathbf{R}^{i+1}\mathbf{R}^i + \mathbf{R}^i\mathbf{R}^{i+1})\right].\end{aligned} \tag{1.8}$$

## 1.3 Area-based centroid and shape tensor

We define the area-based centroid, $\mathbf{R}_A$, of cell $\alpha$, via the cell's first moment of area

$$\mathbf{R}_A = \frac{\int_\alpha \mathbf{R}\, dA}{\int_\alpha dA}, \tag{1.9}$$

where the numerator is the first moment of area, $\mathbf{R}$ represents vectors across the cell area from the origin, $dA$ is an area element and the integral is across the cell interior. This is the classically defined centroid, equivalent to the centre of a best-fit ellipse. The integral can be evaluated using Green's theorem and, assuming the cell is a convex polygon, the formula may be discretised to give

$$\mathbf{R}_A = \frac{1}{6A} \sum_{i=0}^{P-1} [\mathbf{R}^i + \mathbf{R}^{i+1}][\hat{\mathbf{z}} \cdot (\mathbf{R}^i \times \mathbf{R}^{i+1})], \tag{1.10}$$

where $A = \sum_{i=0}^{P-1} \frac{1}{2}\hat{\mathbf{z}} \cdot (\mathbf{R}^i \times \mathbf{R}^{i+1})$ is the area of the polygon and $\hat{\mathbf{z}}$ is a unit vector pointing out of the plane.

To define the area-based shape tensor, let us consider the triangle, $\Delta^i = \{\mathbf{R}_A, \mathbf{R}^i, \mathbf{R}^{i+1}\}$, and define

$$\mathbf{R} = \mathbf{R}_A + a\mathbf{R}^i + b\mathbf{t}^i, \tag{1.11}$$

for $\mathbf{R}$ in $\Delta^i$. The edges of the triangle are then given by

$$b = 0, \qquad 0 < a < 1 \tag{1.12a}$$
$$a = 1, \qquad 0 < b < 1 \tag{1.12b}$$
$$b = a, \qquad 0 < a < 1. \tag{1.12c}$$

Computing the second moment of the triangle, $\Delta^i$, we have

$$\begin{aligned}
\mathsf{S}_{\Delta^i} &= \int_{\Delta^i} (\mathbf{R} - \mathbf{R}_A)(\mathbf{R} - \mathbf{R}_A)\,\mathrm{d}A. \\
&= \int_{\Delta^i} (a\mathbf{R}^i + b\mathbf{t}^i)(a\mathbf{R}^i + b\mathbf{t}^i)\,\mathrm{d}A \\
&= \int_{\Delta^i} \left[a^2\mathbf{R}^i\mathbf{R}^i + ab(\mathbf{R}^i\mathbf{t}^i + \mathbf{t}^i\mathbf{R}^i) + b^2\mathbf{t}^i\mathbf{t}^i\right]\mathrm{d}A.
\end{aligned} \tag{1.13}$$

Taking an area element of the triangle gives

$$\begin{aligned}
\mathrm{d}A &= \left|\frac{\partial(x,y)}{\partial(a,b)}\right| \mathrm{d}a\,\mathrm{d}b \\
&= \left|\hat{\mathbf{z}} \cdot \mathbf{R}^i \times \mathbf{t}^i\right| \mathrm{d}a\,\mathrm{d}b.
\end{aligned} \tag{1.14}$$

Integrating over the triangle, we have

$$\begin{aligned}
\int_{\Delta^i} \mathrm{d}A &= \int_0^1 \mathrm{d}a \int_0^a \mathrm{d}b \left|\hat{\mathbf{z}} \cdot \mathbf{R}^i \times \mathbf{t}^i\right| \\
&= \tfrac{1}{2}\left|\hat{\mathbf{z}} \cdot \mathbf{R}^i \times \mathbf{t}^i\right| \\
&= A^i,
\end{aligned} \tag{1.15}$$

where $A^i$ is the area of $\Delta^i$. Thus

$$\begin{aligned}
\mathsf{S}_{\Delta^i} &= \mathbf{R}^i\mathbf{R}^i \int_0^1 \mathrm{d}a \int_0^a a^2 2A^i\,\mathrm{d}b + \mathbf{t}^i\mathbf{t}^i \int_0^1 \mathrm{d}a \int_0^a b^2 2A^i\,\mathrm{d}b \\
&\quad + (\mathbf{R}^i\mathbf{t}^i + \mathbf{t}^i\mathbf{R}^i) \int_0^1 \mathrm{d}a \int_0^a ab2A^i\,\mathrm{d}b \\
&= A^i\left[\tfrac{1}{2}\mathbf{R}^i\mathbf{R}^i + \tfrac{1}{6}\mathbf{t}^i\mathbf{t}^i + \tfrac{1}{4}(\mathbf{R}^i\mathbf{t}^i + \mathbf{t}^i\mathbf{R}^i)\right] \\
&= \frac{A^i}{6}(\mathbf{R}^{i+1}\mathbf{R}^{i+1} + \mathbf{R}^i\mathbf{R}^i) + \frac{A^i}{12}(\mathbf{R}^{i+1}\mathbf{R}^i + \mathbf{R}^i\mathbf{R}^{i+1}).
\end{aligned} \tag{1.16}$$

Finally, the area-based shape tensor, $\mathsf{S}_A$, can be defined as the sum of the second moments of each sub-triangle:

$$\begin{aligned}
\mathsf{S}_A &= \int_\alpha (\mathbf{R} - \mathbf{R}_A)(\mathbf{R} - \mathbf{R}_A)\,\mathrm{d}A \\
&= \sum_{i=0}^{Z-1} \mathsf{S}_{\Delta^i} \\
&= \sum_{i=0}^{Z-1} \left\{\frac{A^i}{6}(\mathbf{R}^{i+1}\mathbf{R}^{i+1} + \mathbf{R}^i\mathbf{R}^i) + \frac{A^i}{12}(\mathbf{R}^{i+1}\mathbf{R}^i + \mathbf{R}^i\mathbf{R}^{i+1})\right\}.
\end{aligned} \tag{1.17}$$

### 1.4 Cell circularity and orientation

The cell shape tensors, $\mathsf{S}_X$ (for $X \in \{J, P, A\}$), have eigenvalues $\lambda_{X,1} \geq \lambda_{X,2}$. We define the $X$-based cell circularity as

$$C_X = \frac{\lambda_{X,2}}{\lambda_{X,1}}, \tag{1.18}$$

such that $0 \geq C_X \geq 1$ and $C_X$ is larger for more rounded cells. The orientation of $X$-based cell shape, $\theta_X$, may then be defined as the orientation of the eigenvector associated with the largest eigenvalue.

# 2 Predicting division orientation using a mechanical model based on microtubule pulling forces

The geometric shape-based predictions of division were compared against a mechanical shape-based model of cell division, in which astral microtubules are hypothesised to exert pulling forces on the spindle (Minc et al., (2011)). The spindle is assumed to lie in the 2D apical plane of the cell, centred at the cell centroid, $\mathbf{R}_P$ (choosing $\mathbf{R}_P$ to account for non-straight edges). Microtubules (MTs) emanate from the opposed centrosomes of the spindle in a straight line towards the cortex. Anchoring at the cell cortex, the MTs are assumed to exert length-dependent forces towards the centrosome. This leads to a torque on the spindle about the centroid, dependent on the angle, $\phi$, between the MT and the spindle axis. The configuration can be visualised in Supplemental Figure 4B.

Let the spindle, of length $L$, be oriented at angle $\theta \in [0, \pi]$ relative to a reference axis. We create two asters at each end of the spindle, labelled $j = 1, 2$ and positioned at $(-L/2, L/2)$. Each aster has $N$ MTs of length $l_i(\phi, \theta)$, placed at a constant angular density. A MT oriented at angle $\phi$ relative to the spindle axis exerts a length-dependent force of magnitude

$$f_i(\phi, \theta) = l_i^{\beta}(\phi, \theta) \cos \phi \tag{2.1}$$

on the spindle, along the axis of the microtubule. $\beta$ is a variable governing how the force exerted by a MT scales with its length. Correspondingly, the torque exerted on the spindle by the MT is

$$\tau_i(\phi, \theta) = \frac{L}{2} l_i^{\beta}(\phi, \theta) \sin \phi. \tag{2.2}$$

The total torque, $T$, exerted by aster $j$ on the spindle is then given by

$$T_j(\theta) = \int_{-\phi_{\text{max}}}^{\phi_{\text{max}}} \rho \tau_i(\phi, \theta) \, \mathrm{d}\phi \tag{2.3}$$

where $\phi_{\text{max}}$ is the maximum angle that a MT makes relative to the spindle, such that $2\phi_{\text{max}}$ is the angular width of the aster, and $\rho = 2\phi_{\text{max}}/N$ is the constant angular density. The net torque exerted on the spindle is the sum of torques from each aster:

$$T_S(\theta) = T_1(\theta) + T_2(\theta) \tag{2.4}$$

(2.4) can be computed for $\theta \in [0, \pi]$ and the cell's predicted division angle, $\theta_{\text{Minc}}$ is given by the angle at which the net torque is minimised i.e.

$$\theta_{\text{Minc}} = \underset{\theta \in [0, \pi]}{\arg\min} \, T_S(\theta). \tag{2.5}$$

Zeros of (2.4) usually come in pairs, one stable and one unstable, and we choose the stable solution. For the subset of cases where (2.4) has multiple stable zeros (usually occurring in round cells), we choose the solution producing the lowest minimum of the derivative.

The model has parameters $(L, N, \phi_{\max}, \beta)$, corresponding to length of the spindle, number of MTs at each aster, angular extension of the MTs and the force-length scaling parameter. However, previous studies with this model have found that a number of these parameters become redundant beyond a certain value (Minc et al. (2011), Bosveld et al. (2016)). Bosveld et al. (2016) varied the spindle length, $L$, to 1/8 of the experimentally measured value and found no discernible change in the predicted division angles. Following average spindle length measurements by Woolner et al. (2008), we therefore set $L = \sqrt{A/\pi}$, where $A$ is the area of the cell, using the length of the cell fitted to a circle as the default spindle length. Similarly, choosing values of $N > 15$ and $\phi_{\max} > 120$ did not change overall predictions, so we set $N = 100$ and $\phi_{\max} = 180$ for all experiments. Conversely, varying $\beta$ can produce discernible differences, with $\beta = 3$ producing the best prediction for Minc et al. (2011). We therefore followed Minc and colleagues by calculating all predictions for $\beta = 1, 2, 3$. Though predicted division angles differed slightly between these values, we found no significant difference when compared to predictions of division angle relative to the positioning of tricellular junctions (Figures 2I&J and S1G&H).

# 3 The vertex-based model

To model the cells mathematically, we adopt a well-established vertex-based constitutive model (Farhadifar et al., (2007), Ishihara et al., (2012), Nestor-Bergmann et al., (2017)). Under the vertex-based model, forces are obtained by assuming that every cell has a mechanical energy, $\tilde{U}$, of the following form

$$\tilde{U} = \tfrac{1}{2}\tilde{K}\left(\tilde{A} - \tilde{A}_0\right)^2 + \tfrac{1}{2}\tilde{\Gamma}\tilde{L}^2 + \tfrac{1}{2}\tilde{\Lambda}\tilde{L}, \tag{3.1}$$

where $\tilde{A}$ is the area of a cell, $\tilde{L}$ is the perimeter, $\tilde{A}_0$ is the preferred cell area, $\tilde{K}$ is a bulk stiffness and $(\tilde{\Lambda}, \tilde{\Gamma})$ are model parameters representing contractility of the cell periphery. We nondimensionalise, scaling length scales on $\sqrt{\tilde{A}_0}$, using

$$\tilde{A} = \tilde{A}_0 A, \quad \tilde{L} = \sqrt{\tilde{A}_0}L, \quad \tilde{U} = \tilde{K}\tilde{A}_0^2 U, \quad \tilde{t} = \eta t/(\tilde{K}\sqrt{\tilde{A}_0}), \tag{3.2}$$

where $\tilde{t}$ is time and $\eta$ is a viscous drag coefficient. Thus the nondimensional mechanical energy, $U$, may be written

$$U = \tfrac{1}{2}\left(A_\alpha - 1\right)^2 + \tfrac{1}{2}\Gamma(L_\alpha - L_0)^2 - U_0 \tag{3.3}$$

where $U_0 = \Lambda^2/4\Gamma^2$ is a constant that may be discarded as the dynamics are driven by energy gradients and we have written $L_0 = -\Lambda/2\Gamma$ which defines an effective preferred cell perimeter. We are left with two nondimensional parameters

$$\Gamma = \frac{\tilde{\Gamma}}{\tilde{K}\tilde{A}_0}, \qquad \Lambda = \frac{\tilde{\Lambda}}{\tilde{K}\tilde{A}_0^{3/2}}, \tag{3.4}$$

which have previously been fitted to *Xenopus laevis* animal cap explants with values $(\Lambda, \Gamma) = (-0.259, 0.172)$ (see Nestor-Bergmann et al., (2017) for details on the fitting procedure). We assume that the cells have identical physical properties, such that $(\Lambda, \Gamma)$ do not vary across the cells.

Under this constitutive equation, a cell can be mechanically characterised by its stress tensor, $\boldsymbol{\sigma}$, given by (Nestor-Bergmann et al., (2017) and Sugimura et al., (2016))

$$\boldsymbol{\sigma} = \overline{\boldsymbol{\sigma}} + \boldsymbol{\sigma}' - \frac{1}{2}\dot{\mathsf{S}}_J \quad (3.5)$$

where $\dot{\mathsf{S}}_J$ is the time derivative of the junctional shape tensor (1.2), which gives the dissipative stress acting when cells are out of equilibrium and arises from a simple choice of drag law on the vertices. We have written the elastic stress as a sum of isotropic and deviatoric contributions, with the former given by

$$\overline{\boldsymbol{\sigma}} = -P^{\text{eff}}\mathsf{I}, \qquad P^{\text{eff}} = A - 1 + \frac{\Gamma L^2}{2A} - \frac{\Lambda L}{4A}, \quad (3.6)$$

where $\mathsf{I}$ is the identity and $P^{\text{eff}}$ is an effective cell pressure. The traceless contribution is

$$\boldsymbol{\sigma}' = \frac{\Gamma(L - L_0)}{A}\left(\frac{L}{2}\mathsf{I} - \sum_{j=0}^{Z-1} l^j \hat{\mathbf{t}}^j \hat{\mathbf{t}}^j\right) \quad (3.7)$$

where the cell has $Z$ tricellular junctions, labelled anticlockwise $j = 0, 1, \dots, Z-1$, and $\hat{\mathbf{t}}^j$ is the unit tangent connecting tricellular junctions $j$ and $j+1$ and $l^j$ is the length of that edge.

Assuming the cells are in equilibrium ($\dot{\mathsf{S}}_J = 0$), we can focus on the elastic contribution. Here the stress tensor will have eigenvalues

$$\sigma_1 = -P^{\text{eff}} + \xi \qquad \sigma_2 = -P^{\text{eff}} - \xi \quad (3.8)$$

such that $|\sigma_1| \geq |\sigma_2|$. $-P^{\text{eff}}$ is the eigenvalue of $\overline{\boldsymbol{\sigma}}$, by which we characterise the magnitude of isotropic stress. $\pm\xi$ are the eigenvalues of $\boldsymbol{\sigma}'$, with $\xi = \sqrt{\det(\boldsymbol{\sigma}')}$, representing the magnitude of shear stress. We term the eigenvector, $\boldsymbol{\sigma}_1$, associated with $\sigma_1$ the principal axis of stress. For $P^{\text{eff}} > 0$ ($< 0$) we have a cell dominantly under tension (compression). If $|\xi| > |P^{\text{eff}}|$, the cell is in a state of shear where the principal components of stress have opposite signs ($\sigma_1\sigma_2 < 0$). Supplemental Figure 4C outlines the four possible cases of cell stress, based on the signs of the eigenvalues.

# 4 Nondimensionalising the experimental data

The nondimensionalisation procedure uses the scalings on area and perimeter given in (3.2). In order to nondimensionalise the areas and perimeters from the experimental data, we must estimate the value of $\tilde{A}_0$ for the *Xenopus laevis* animal cap cells. Under (3.5), the isotropic component of the nondimensional tissue stress, $P_{\text{tis}}$, of a monolayer in equilibrium may be expressed as an area-weighted sum of the effective pressures (Nestor-Bergmann et al., (2017))

$$P_{\text{tis}} = \frac{1}{A_{\text{tis}}} \sum_{\alpha=1}^{N_c} A_\alpha P_\alpha^{\text{eff}} \quad (4.1)$$

where $A_{\text{tis}}$ is the total surface area of the monolayer and the summation is over all cells labelled $\alpha = 1, \dots, N_c$. Under conditions of zero external loading, $P_{\text{tis}} = 0$ and, substituting the scalings for area and perimeter in (3.2), we may write

$$\sum_{\alpha=1}^{N_c} \frac{\tilde{A}_\alpha}{\tilde{A}_0}\left(\frac{\tilde{A}_\alpha}{\tilde{A}_0} - 1 + \frac{\Gamma \tilde{L}_\alpha^2}{2\tilde{A}_\alpha} + \frac{\Lambda \tilde{L}_\alpha \sqrt{\tilde{A}_0}}{4\tilde{A}_\alpha}\right) = 0. \quad (4.2)$$

Thus, given previously fitted values of $(\Lambda, \Gamma)$ and measurements of experimental areas, $\tilde{A}_\alpha$, and perimeters, $\tilde{L}_\alpha$, we can select the value of $\tilde{A}_0$ which best satisfies (4.2); see supplementary movie 1 for an example of the process). Given that the mean cell area varies between experiments (due to each embryo being at a slightly different stage of development when imaged) we fit a specific $\tilde{A}_0$ to every experiment. The implicit assumption – that the sample of experimental cells measured is under zero net peripheral stress – highlights that the results will be more accurate as the sample size increases.

Supplemental Figure 4D shows the mean cell areas, $\langle \tilde{A} \rangle$, from 10 experiments plotted against the fitted values of $\tilde{A}_0$. We see a strikingly linear relationship, indicating that the preferred area is well predicted by the mean area under our fitting process, where $\tilde{A}_0 \approx 4.45 \langle \tilde{A} \rangle$. These data also emphasise the variability in mean area across experiments, due the animal caps being at slightly different stages of development, indicating that each experiment requires a unique $\tilde{A}_0$. Supplemental Figure 4E shows the equivalent plot for myosin II MO cells, in which we see the cells have a significantly larger preferred area but $\tilde{A}_0 \approx 4.45 \langle \tilde{A} \rangle$ holds.

We can then infer the relative nondimensional effective pressure, for example, of the experimental cells by epressing nondimensional areas and perimeters in terms of the fitted $\tilde{A}_0$

$$P^{\text{eff}} = \frac{\tilde{A}}{\tilde{A}_0} - 1 + \frac{\Gamma \tilde{L}^2}{2\tilde{A}} + \frac{\Lambda \tilde{L} \sqrt{\tilde{A}_0}}{4\tilde{A}}, \tag{4.3}$$

where we have dropped the $\alpha$ subscripts. In the absence of a measurement of the stiffness parameter, $K$, (4.3) reveals relative stresses within and between cells in tissue, but not their absolute magnitude.

Supplemental Figures 4F&G demonstrate the relationship between cell area, perimeter and $P^{\text{eff}}$. Strikingly, we find that $P^{\text{eff}}$ values calculated from both simulated (Supplemental Figure 4F) and experimental (Supplemental Figure 4G) data lie close to those predicted for perfect polygons, which have an exact relationship between area and perimeter (curves plotted in Supplemental Figures 4F&G). This indicates that the magnitude of isotropic cell stress is well predicted by area and number of sides. However, our results also demonstrate that cell area alone is not a perfect indicator of relative effective cell pressure. We find many cells have a negative (positive) value of $P^{\text{eff}}$, suggesting that they are under compression (tension), but have an area larger (smaller) than the mean (see blue cells above mean area and red cells below mean area in Supplemental Figure 4H). Examples of these special cases are given in Supplemental Figure 4I, where it is apparent that these cells do not look especially unusual.
